# Acquired Chemoresistance in Colorectal Cancer: Therapy-Induced Signalling Adaptation, Cellular Plasticity and Mechanism-Guided Therapeutic Strategies

**DOI:** 10.3390/cells15191776

**Published:** 2026-09-29

**Authors:** Dorota Bartusik-Aebisher, Izabella Wilk, Blassan P. George, David Aebisher

**Affiliations:** 1 Department of Biochemistry and General Chemistry, Faculty of Medicine, Collegium Medicum, University of Rzeszów, 35-959 Rzeszów, Poland; dbartusikaebisher@ur.edu.pl; 2 English Division Science Club, Faculty of Medicine, Collegium Medicum, University of Rzeszów, 35-959 Rzeszów, Poland; iw135750@stud.ur.edu.pl; 3 Laser Research Centre, Faculty of Health Sciences, University of Johannesburg, Doornfontein, P.O. Box 17011, Johannesburg 2028, South Africa; blassang@uj.ac.za; 4 Department of Photomedicine and Physical Chemistry, Faculty of Medicine, Collegium Medicum, University of Rzeszów, 35-959 Rzeszów, Poland

**Keywords:** colorectal cancer, acquired chemoresistance, 5-fluorouracil, oxaliplatin, irinotecan, Wnt/β-catenin signalling, Notch signalling, Hippo/YAP1 pathway, cancer stem cells, tumour microenvironment

## Abstract

**Highlights:**

**What are the main findings?**

- Resistance to 5-fluorouracil (5-FU), oxaliplatin and irinotecan involves distinct drug-proximal mechanisms, while adaptive cellular states and microenvironment-mediated processes can support treatment survival.
- Evidence linking Wnt/β-catenin, Notch and Hippo/Yes-associated protein 1 (YAP1) signalling to chemoresistance is predominantly preclinical and context-dependent, with stronger direct pathway-specific evidence for 5-FU and oxaliplatin than for irinotecan.

**What are the implications of the main findings?**

- Drug-specific resistance mechanisms should be distinguished from reversible treatment-tolerant states and broader microenvironmental processes when interpreting chemoresistance.
- Biomarker-selected, mechanism-matched therapeutic strategies and prospective clinical validation should be prioritised to advance translation of resistance-directed approaches.

**Abstract:**

The effectiveness of chemotherapy in colorectal cancer (CRC) is significantly reduced by acquired chemoresistance to 5-fluorouracil (5-FU), oxaliplatin and irinotecan. This structured narrative review synthesises mechanistic, preclinical, translational and clinical evidence underlying resistance to these agents. Distinct drug-specific molecular mechanisms can underlie acquired chemoresistance, whereas shared adaptive survival processes support treatment persistence and may, in some settings, contribute to the emergence of stable acquired resistance. 5-FU resistance is associated with factors such as altered thymidylate synthase activity, drug catabolism, nucleotide metabolism and apoptosis avoidance, whereas resistance to oxaliplatin involves enhanced deoxyribonucleic acid (DNA) repair, detoxification, reduced platinum accumulation and metabolic adaptation. Irinotecan resistance can involve reduced *TOP1* expression or *TOP1* mutations, altered SN-38 metabolism, increased drug efflux and enhanced tolerance of replication-associated stress. Alongside these drug-proximal mechanisms, drug-tolerant persister states, stem-like programmes, epithelial–mesenchymal plasticity and multidrug-efflux mechanisms can support treatment survival and tumour repopulation. Evidence linking chemoresistance with Wnt/β-catenin, Notch and Hippo/Yes-associated protein 1 (YAP1) signalling is predominantly preclinical and varies according to treatment context, with stronger direct pathway-specific evidence for 5-FU and oxaliplatin than for irinotecan. This distinction allows direct drug-specific associations to be differentiated from broader roles in adaptive survival, cellular plasticity and tumour repopulation after treatment. Cancer-associated fibroblasts, immune and cytokine signalling, extracellular vesicles, hypoxia and treatment-induced remodelling of the tumour microenvironment can all further influence tumour heterogeneity and non-cell-autonomous resistance. Residual disease, resistant cellular states and therapeutically relevant molecular dependencies may be detected or characterised using circulating tumour DNA, pathway-associated biomarkers, patient-derived organoids and emerging resistance-profiling technologies. These findings show that interactions between drug-specific molecular escape mechanisms, adaptive cellular states and the tumour microenvironment contribute to heterogeneous patterns of treatment survival and acquired chemoresistance. The development of molecularly targeted and mechanism-guided strategies for overcoming resistance in CRC may be supported by defining these mechanisms and their associated biomarkers.

## 1. Introduction

Colorectal cancer (CRC) represents a major global health burden and remains one of the three most frequently diagnosed cancers worldwide. CRC is the second leading cause of cancer-related death worldwide, after lung cancer. These epidemiological data emphasise the high incidence and mortality of CRC [1,2]. The age distribution of CRC is also changing, with increasing incidence among younger adults in many regions [3]. Fluoropyrimidine-based combination chemotherapy remains a major component of systemic treatment for CRC. In advanced disease, commonly used regimens include folinic acid, 5-fluorouracil (5-FU) and oxaliplatin (FOLFOX) and folinic acid, 5-FU and irinotecan (FOLFIRI). FOLFOX and FOLFIRI were established as standard chemotherapy options for metastatic CRC by randomised clinical trials [4,5]. Capecitabine is an oral prodrug of 5-FU and is also used as the fluoropyrimidine component of regimens such as capecitabine plus oxaliplatin (CAPOX) [6]. Depending on the molecular profile and primary tumour location, these chemotherapy regimens may also be combined with targeted agents. This includes the anti-vascular endothelial growth factor (VEGF) antibody bevacizumab or, in RAS wild-type left-sided metastatic CRC, the anti-epidermal growth factor receptor (EGFR) antibodies cetuximab and panitumumab [7]. Although metastatic CRC may be controlled by these regimens initially, tumour progression may occur during treatment or after an initial response to treatment [8]. Recurrence after curative-intent surgery occurs in both stage II and stage III CRC. However, the risk of cancer recurrence after curative-intent surgery is higher in stage III disease [9]. These patterns demonstrate that chemoresistance remains a serious clinical challenge. Chemoresistant metastases can continue to grow despite systemic treatment in metastatic CRC and may spread to additional sites. Continued disease progression can worsen symptoms, impair the function of affected organs, reduce the remaining treatment options, and ultimately contribute to poorer survival, as these metastases no longer adequately respond to chemotherapy [8]. Understanding how these treatments exert their cytotoxic effects is important for understanding how resistance develops. The cytotoxic agents considered in this review primarily impair nucleic acid synthesis or deoxyribonucleic acid (DNA) integrity and activate cellular stress responses. 5-FU inhibits thymidylate synthase, while fluorinated metabolites are incorporated into ribonucleic acid (RNA) and DNA. Through these processes, 5-FU impairs the replication and metabolism of nucleic acids [10,11]. Oxaliplatin causes the formation of platinum-DNA adducts and crosslinks. Their formation alters the DNA helix and blocks replication. Transcription also becomes limited, and many stress-response pathways become activated [12,13]. Irinotecan generates the metabolite 7-ethyl-10-hydroxycamptothecin (SN-38), which inhibits the enzyme topoisomerase I. SN-38 also converts replication-associated lesions into cytotoxic breaks in DNA by stabilising cleavage complexes [14,15]. The cytotoxic effects of these agents also depend on intact DNA-damage responses, cell-cycle checkpoints and apoptotic signalling. Defects in these pathways can permit survival despite chemotherapy-induced damage and thereby contribute to resistance [16].

**Figure 1:**
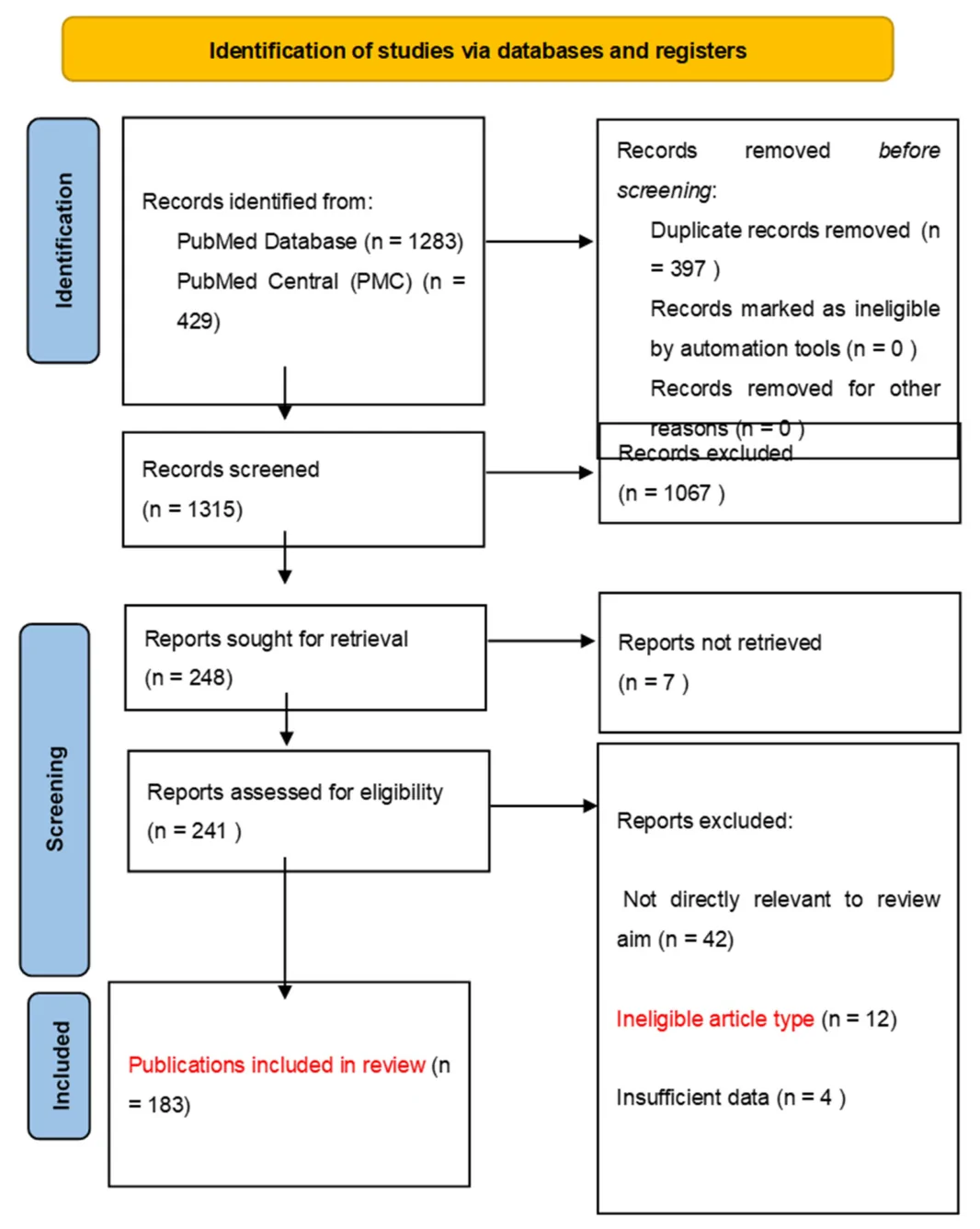
Study-selection flow diagram adapted from PRISMA 2020 to transparently document literature identification, screening, eligibility assessment and inclusion in this structured narrative review.

In CRC, acquired chemoresistance may reflect both selection of pre-existing resistant populations and treatment-induced adaptive survival states. Reversible drug-tolerant states are therefore distinguished in this review from stable acquired resistance, with operational definitions provided in Section 2.3 and Section 2.4. Broad overviews of signalling pathways and complex mechanisms involved in colorectal cancer treatment resistance have been offered by recent reviews [17,18]. This review applies an evidence-weighted, drug-context-specific approach to distinguish drug-proximal mechanisms of resistance from broader adaptive cellular and tumour-microenvironmental processes that may contribute to treatment survival across multiple therapeutic contexts. The analysis focuses on acquired chemoresistance to 5-FU, oxaliplatin and irinotecan, with Wnt/β-catenin, Notch and Hippo/Yes-associated protein 1 (YAP1) signalling evaluated according to the specific drug context and strength of supporting evidence. This structured narrative review used a predefined literature search and screening process to support transparent identification of the evidence informing the synthesis, as summarised in Figure 1.

## 2. Methods

This manuscript was designed as a structured narrative review rather than a systematic or scoping review. A predefined database search, explicit eligibility criteria, independent screening by two reviewers and a PRISMA-adapted study-selection flow diagram were used to improve the transparency and reproducibility of literature identification and selection. The review aimed to provide an interpretive, evidence-weighted synthesis of heterogeneous mechanistic, preclinical, translational and clinical evidence on acquired chemoresistance. Findings were therefore synthesised narratively, and no meta-analysis or pooled effect estimates were performed (Table S1. Database-specific search strategies used for literature identification, Table S2. Structured evidence appraisal of all publications included in the review).

### 2.1. Search Strategy and Scope

PubMed and PubMed Central (PMC) were searched, with the final searches conducted on 20 August 2026. PubMed was used as the principal bibliographic database, while PMC was searched as a supplementary full-text source to identify potentially relevant studies that might not have been retrieved through the PubMed search strategy. Given the substantial overlap between PubMed and PMC, records retrieved from both sources were combined and duplicate records were removed before screening.

Separate topic-focused searches were performed for PubMed and PMC. The Boolean operator AND was used to combine major search concepts, whereas OR was used to combine synonymous or related terms. Database field tags included Medical Subject Headings (MeSH) Terms, Supplementary Concept and All Fields, as applicable to the individual search. No language, study-design, publication-type or publication-date filters were applied at the database-search stage. All potentially eligible records identified up to the final search date were considered. The publications ultimately included in the review were published between 1998 and July 2026. Complete database-specific search strings and applied filters are provided in Supplementary Table S1.

### 2.2. Eligibility Criteria and Evidence Synthesis

Primary experimental, translational and clinical studies were eligible if they investigated CRC or CRC-derived models and provided relevant evidence on acquired resistance or treatment tolerance involving 5-fluorouracil (5-FU), oxaliplatin or irinotecan. No specific study-design restriction was imposed beyond relevance to the review scope. No language restriction was applied during eligibility assessment. Studies addressing associated resistance mechanisms, molecular targets, candidate biomarkers or strategies for overcoming resistance were also eligible.

Relevant review articles were included to provide mechanistic, methodological or clinical context and contributed to the screened evidence set. Studies were excluded if they were not directly relevant to the review aim, represented an unsuitable article type or provided insufficient relevant data. Evidence was organised according to drug-specific mechanisms, shared cellular mechanisms, signalling pathways and tumour-microenvironmental mechanisms. A limited number of non-CRC primary studies were retained solely as explicitly labelled proof-of-principle or contextual evidence and were not used to establish CRC-specific resistance mechanisms or increase evidence grades.

### 2.3. Study Selection and Data Extraction

Two reviewers independently screened titles and abstracts and assessed the full texts of potentially eligible publications. Disagreements regarding study inclusion were resolved by discussion, and no automation tools were used.

Data were extracted using a standardised framework comprising study design and experimental model, chemotherapy or treatment context, resistance phenotype, mechanism or signalling pathway investigated, functional perturbation or rescue experiments, validation in complementary models, human or clinical validation, and principal findings relevant to chemoresistance. Data were initially extracted by one reviewer and subsequently verified against the original publications by a second reviewer. Discrepancies or uncertainties were resolved by discussion and consensus before inclusion in the narrative synthesis and evidence appraisal.

For synthesis, resistance phenotypes were classified as stable acquired resistance, reversible drug-tolerant/persister states, stem-like phenotypes or functional cancer stem cell (CSC) states. Stable acquired resistance was assigned when reduced drug sensitivity persisted after treatment withdrawal and rechallenge or was demonstrated in an established resistant model. Findings from acute or short-term drug exposure were classified as treatment-tolerant or treatment-surviving unless persistent resistance was demonstrated. Stemness-associated marker expression or transcriptional programmes were described as stem-like, whereas CSC terminology was reserved for studies providing functional evidence of tumour-initiating capacity.

Study-selection numbers and reasons for full-text exclusion are summarised in Figure 1. Seven of the 248 reports sought for retrieval could not be obtained because their full texts were unavailable through the searched databases, publisher websites or available institutional access at the time of the review. Overall, 183 publications were included.

### 2.4. Structured Evidence Appraisal

Because the included literature comprised heterogeneous cell-line, organoid, animal, translational and clinical studies, a single design-specific risk-of-bias tool was not considered appropriate across all study types. Instead, the strength of evidence supporting the principal mechanistic and therapeutic conclusions was assessed using a structured evidence-grading framework.

Evidence was evaluated according to: (i) direct relevance to acquired chemoresistance to 5-FU, oxaliplatin or irinotecan in CRC; (ii) functional demonstration of the proposed mechanism, including genetic or pharmacological perturbation, sensitisation or rescue where applicable; (iii) validation across complementary experimental models, including cell lines, patient-derived organoids (PDOs) or in vivo models; and (iv) evidence from human tumour samples or clinical studies.

Evidence was classified as strong, moderate, limited or indirect. Strong evidence required direct support for the proposed resistance mechanism or therapeutic strategy in human or clinical material together with concordant mechanistic evidence from experimental models. Moderate evidence required direct mechanistic support with functional validation and confirmation across complementary CRC models, including organoid or in vivo evidence. Limited evidence was assigned when support arose predominantly from cell-line or single-model studies or when functional validation was incomplete. Indirect evidence was assigned when the proposed relationship was extrapolated from related biological or treatment contexts without direct demonstration of the specific drug–resistance mechanism in CRC. Where no direct evidence supporting a proposed pathway–resistance relationship was identified, the relationship was classified as not established.

Pathway–drug relationships were additionally classified by mechanistic directness as direct causal, associative, indirect/shared adaptive-survival or not established. Direct causal classification required functional pathway manipulation that altered sensitivity to the specified drug; associative evidence reflected pathway activation or expression linked to resistance without sufficient causal validation, whereas indirect/shared adaptive-survival evidence referred to pathway effects on treatment tolerance, plasticity or residual-cell survival without demonstrated regulation of the drug-proximal mechanism. Mechanistic directness was considered separately from overall evidence strength.

Review articles were used for mechanistic, methodological or clinical context but were not used independently to increase evidence grades. The framework was intended to support transparent comparison of evidence strength and translational maturity rather than to constitute a formal GRADE certainty-of-evidence assessment. Study-level application of this framework is provided in Supplementary Table S2; publications used only for background or contextual synthesis are identified as “not independently graded”.

## 3. Drug-Specific and Shared Chemoresistance Mechanisms

### 3.1. 5-Fluorouracil Resistance

5-FU remains an essential part of CRC chemotherapy in fluoropyrimidine-based regimens. Inhibition of thymidylate synthase (TYMS) is a major mechanism of 5-FU cytotoxicity. TYMS inhibition depletes deoxythymidine monophosphate and downstream thymidine nucleotide pools required for DNA synthesis, while fluorinated metabolites are incorporated into ribonucleic acid (RNA) and DNA. This contributes to the cytotoxic activity of the drug by disrupting the metabolism of nucleic acids and by causing cell stress [11,19]. TYMS overexpression or amplification of the *TYMS* gene is a leading mechanism of 5-FU resistance. Elevated TYMS expression can maintain thymidylate synthesis despite 5-FU exposure, thereby reducing drug sensitivity. Research has shown that reduced sensitivity to fluoropyrimidines in CRC is associated with increased expression of TYMS [16,20]. Increased drug catabolism can also result in resistance to 5-FU. 5-FU is catabolised by dihydropyrimidine dehydrogenase (DPD), encoded by *DPYD*. DPD is the initial rate-limiting enzyme in 5-FU catabolism and is a major determinant of systemic and intratumoural drug exposure. Upregulation of DPD in CRC cells can enhance 5-FU catabolism, reduce intracellular drug exposure and thereby promote resistance [21,22,23]. Altered nucleotide metabolism can also contribute to 5-FU resistance. Reprogramming of pyrimidine metabolism or nucleotide-salvage pathways may maintain nucleotide availability for DNA synthesis and repair, thereby reducing dependence on TYMS-mediated de novo thymidylate synthesis [11,24]. Defective p53-dependent apoptotic signalling may also contribute to 5-FU resistance. 5-FU-induced nucleic-acid damage can activate checkpoint and apoptotic signalling, and defective p53-dependent responses may permit CRC-cell survival despite this damage [16]. Supporting proof-of-principle evidence from a non-CRC model has also demonstrated p53-dependent modulation of the response to 5-FU [25], while CRC-specific evidence further supports the relationship between p53 dysfunction and reduced 5-FU sensitivity [26]. A 2026 study identified EHMT2 as a candidate mediator of acquired 5-FU resistance in CRC. EHMT2 inhibition restored 5-FU sensitivity through PPM1B upregulation in resistant cells, xenografts and patient-derived organoids [27]. A 2025 study also linked UGDH to 5-FU resistance through reactive oxygen species (ROS)-dependent phosphoinositide 3-kinase/protein kinase B (PI3K/AKT)–EEF1A2–PRDX1 signalling, supporting it as another potential molecular target [28].

### 3.2. Oxaliplatin Resistance

CRC chemotherapy with FOLFOX often involves oxaliplatin, a third-generation platinum drug. Oxaliplatin exerts its cytotoxic activity primarily through the formation of platinum–DNA adducts, including intra- and interstrand crosslinks. These changes alter the DNA structure and block its replication and transcription. Checkpoint activation and CRC cell death are eventually triggered by these events [29,30]. Enhanced DNA repair, particularly through the nucleotide excision repair (NER) pathway, can contribute to oxaliplatin resistance. ERCC excision repair 1, an endonuclease non-catalytic subunit (ERCC1), is a component of this pathway that helps to remove DNA lesions caused by oxaliplatin. Many studies have linked high ERCC1 expression with low sensitivity to oxaliplatin and a poor response to treatment [31,32,33]. A 2026 preclinical study identified DNA ligase 1 (LIG1) as a candidate DNA-repair-associated determinant of oxaliplatin resistance. High LIG1 expression was associated with reduced chemosensitivity. Targeting LIG1 reduced DNA repair and enhanced oxaliplatin sensitivity in CRC cells, drug-tolerant persister (DTP) cells, organoids and in vivo models [34]. Elevated glutathione-dependent detoxification can contribute to oxaliplatin resistance through conjugation and sequestration of platinum species, thereby decreasing the fraction available to form cytotoxic DNA adducts [35,36,37]. Altered platinum transport and intracellular accumulation may also influence oxaliplatin response. Copper transporter 1 (CTR1, encoded by *SLC31A1*) is involved in oxaliplatin transport in CRC cells; however, the available CRC evidence does not establish reduced CTR1 expression or function as a causal mechanism of acquired oxaliplatin resistance [38,39]. Lastly, mitochondrial and redox adaptations may contribute to oxaliplatin resistance by altering oxidative metabolism, ROS handling and apoptotic susceptibility. Recent studies have associated oxaliplatin response with oxidative phosphorylation states and cellular ROS handling. These findings support a contributory role for mitochondrial and redox adaptation in some oxaliplatin-resistant CRC models [40,41].

### 3.3. Irinotecan Resistance

Irinotecan is an important part of FOLFIRI therapy. SN-38, the active metabolite of irinotecan, exerts cytotoxic activity by stabilising covalent TOP1–DNA cleavage complexes. Collision of replication forks with these complexes generates replication-associated DNA damage, including potentially lethal DNA strand breaks [42]. Reduced TOP1 expression or mutations in *TOP1* can therefore decrease sensitivity to irinotecan by reducing or modifying the drug target. Reduced TOP1 expression has been observed in irinotecan-resistant CRC models. In addition, decreased drug sensitivity can result from *TOP1* missense mutations. *TOP1* missense mutations may alter drug–target interactions or cleavage-complex formation, thereby reducing irinotecan sensitivity [43,44,45]. Drug efflux is another known mechanism of resistance. ABCB1- and ABCG2-mediated transport can reduce intracellular exposure to irinotecan and/or SN-38, depending on the transporter and substrate context, thereby contributing to reduced drug sensitivity. ABCG2 expression has been associated with reduced irinotecan response in CRC, while ABCB1-mediated efflux can similarly decrease intracellular drug exposure [43,46]. UGT1A1-mediated glucuronidation converts SN-38 to the less active SN-38 glucuronide (SN-38G) and is primarily a pharmacokinetic determinant of active-metabolite exposure rather than an established tumour-intrinsic mechanism of acquired irinotecan resistance [47,48]. TOP1 status and transporter activity may provide mechanistic information on resistance, whereas UGT1A1 should primarily be interpreted in the context of SN-38 metabolism rather than as a tumour-specific resistance marker.

CRC cells can also survive irinotecan-induced replication stress by reinforcing checkpoint and replication-fork protection programmes. Despite persistent TOP1-associated lesions, irinotecan sensitivity can be reduced by ataxia telangiectasia and Rad3-related (ATR)/checkpoint kinase 1 (CHK1) signalling and related stress-response mechanisms. These adaptive responses protect CRC cells from irinotecan-induced damage and have been shown in experimental CRC models [49,50]. A 2025 study reported that FBXW7 deficiency promoted irinotecan resistance through stabilisation of the anti-apoptotic protein MCL1. MCL1 inhibition restored irinotecan sensitivity in FBXW7-deficient CRC models, supporting this axis as a potential resistance-directed target [51].

### 3.4. Cross-Resistance and Shared Mechanisms

**Figure 2:**
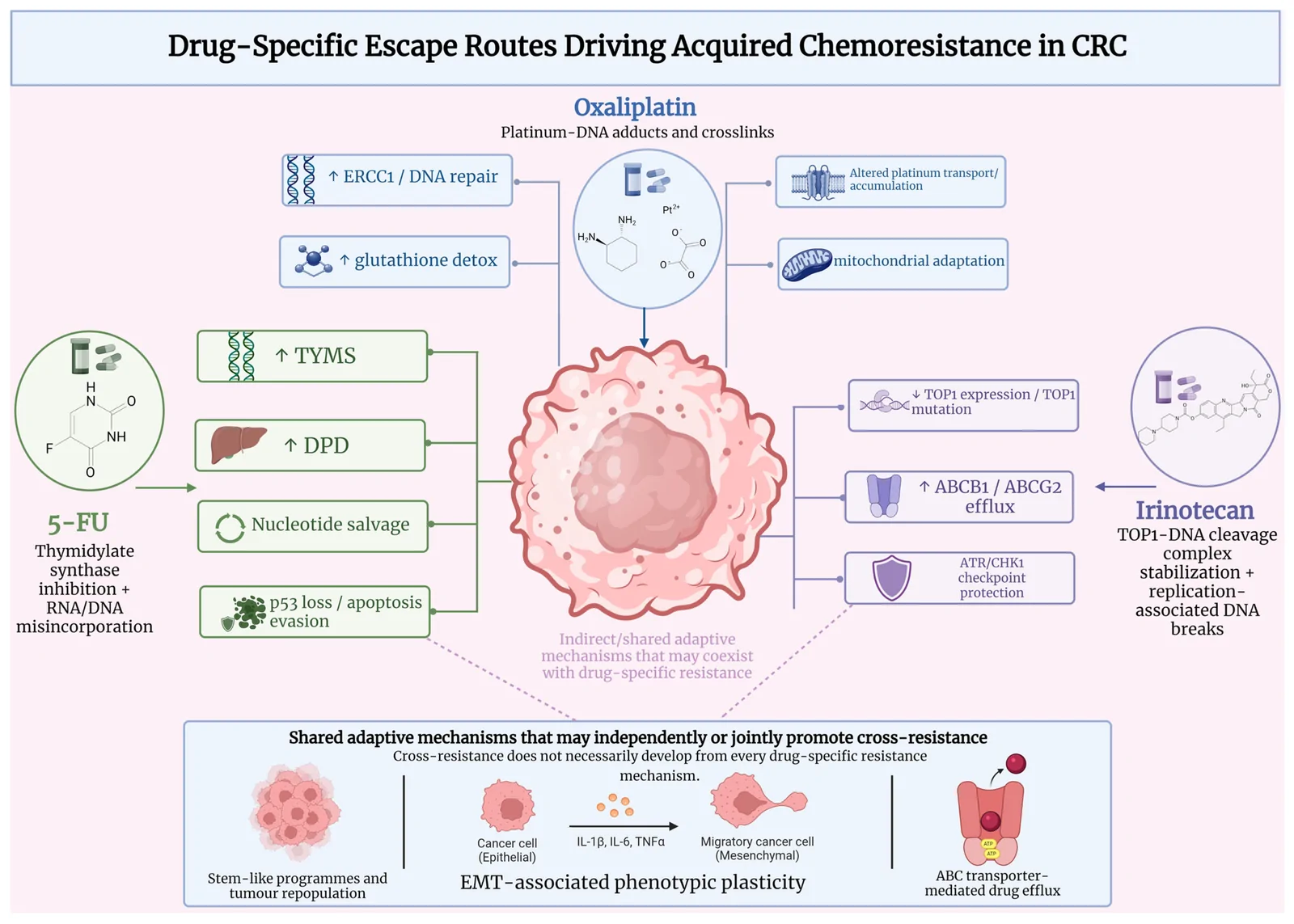
Drug-specific and shared mechanisms of acquired chemoresistance in colorectal cancer. Resistance to 5-fluorouracil, oxaliplatin and irinotecan involves distinct drug-proximal mechanisms, whereas stem-like states and, where functionally demonstrated, CSC-associated survival, epithelial–mesenchymal transition (EMT)-associated phenotypic plasticity and ABC-transporter-mediated drug efflux may contribute across multiple treatment contexts. Drug-proximal mechanisms are presented separately from broader shared adaptive mechanisms, and the dashed connections indicate possible coexistence or indirect association rather than an obligatory causal sequence. Created in BioRender. Wilk, I. (2026) https://BioRender.com/wai004z, accessed on 26 August 2026.

In addition to drug-specific mechanisms, CRC cells may acquire shared phenotypes that reduce sensitivity across multiple treatment contexts. ABCB1/ABCG2-mediated drug efflux, stem-like cellular states and epithelial–mesenchymal plasticity are among the most consistently implicated features (Figure 2) [17,18,52,53,54,55,56,57,58,59]. Increased ATP-binding cassette (ABC) transporter activity can reduce intracellular drug accumulation and thereby contribute to multidrug resistance [53,54]. Epithelial–mesenchymal plasticity may also be accompanied by changes in stemness-associated programmes and transporter activity [57,58,59].

## 4. Wnt/β-Catenin Signalling

### 4.1. Canonical Wnt/β-Catenin Signalling in CRC

**Figure 3:**
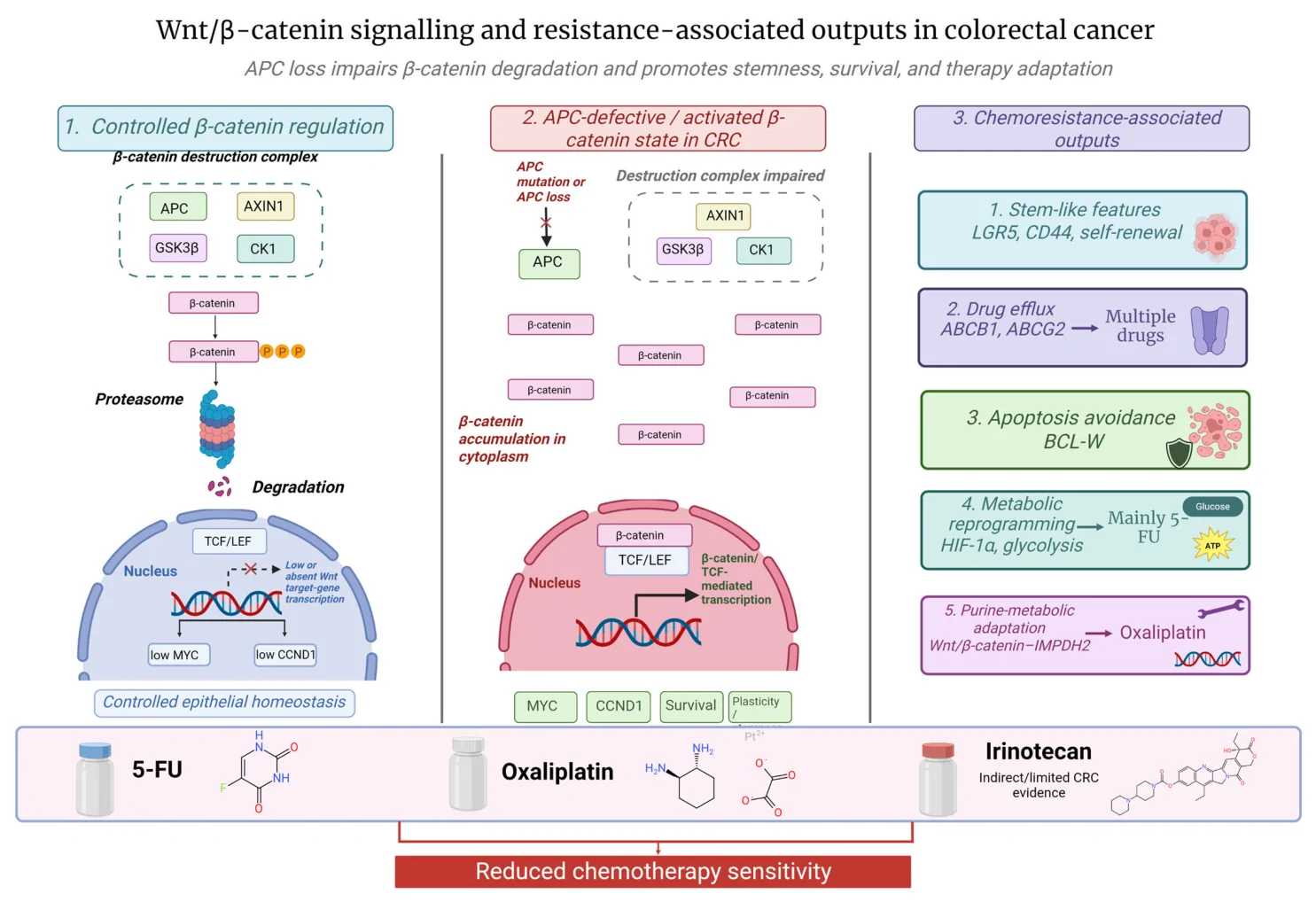
Wnt/β-catenin signalling and resistance-associated outputs in colorectal cancer. APC loss and impaired β-catenin degradation represent the established canonical signalling mechanism leading to nuclear β-catenin/TCF–LEF transcription. Downstream chemoresistance-associated outputs vary in mechanistic directness. Direct drug-specific preclinical evidence is strongest for 5-FU-associated metabolic adaptation and oxaliplatin-associated resistance mechanisms, whereas stem-like features, drug efflux and apoptosis avoidance include broader associative or shared adaptive-survival relationships whose causal relevance varies by treatment context. Direct evidence for Wnt-mediated irinotecan resistance remains limited. Accordingly, graphical inclusion of an output does not imply equivalent causal evidence across all mechanisms. Created in BioRender. Wilk, I. (2026) https://BioRender.com/dgwjoqj.

In conventional CRC, canonical Wnt/β-catenin signalling is most commonly activated by loss-of-function alterations in the *APC* gene. *APC* loss is an early driver event in the adenoma–carcinoma sequence [60,61]. These mutations impair the β-catenin destruction complex, allowing β-catenin accumulation and signalling [60,61]. Normally, APC works with axis inhibition protein 1 (AXIN1), glycogen synthase kinase 3 beta (GSK3β) and casein kinase 1 (CK1) to phosphorylate β-catenin. Once it is phosphorylated, β-catenin is tagged for proteasomal degradation (Figure 3). When APC function is lost, β-catenin escapes efficient degradation, accumulates in the cytoplasm and translocates to the nucleus, where it interacts with T-cell factor (TCF)/lymphoid enhancer factor (LEF) transcription factors to activate genes involved in proliferation and survival [61,62]. Nuclear β-catenin/TCF–LEF signalling increases the transcription of target genes including *MYC*, which links *APC* loss to enhanced anabolic activity and cell-cycle progression [63]. The *CCND1* gene, which encodes cyclin D1, is also upregulated by β-catenin/TCF signalling. Cyclin D1 promotes progression through G1/S and supports uncontrolled cell proliferation in transformed colonic epithelium [64]. Nuclear β-catenin expression has been associated with MYC expression in colorectal adenomas [65].

### 4.2. Wnt-Mediated Mechanisms of Chemoresistance

Wnt/β-catenin signalling can support chemoresistance through several mechanistically distinct outputs, including the maintenance of stem-like states, ABC-transporter-mediated drug efflux, apoptosis avoidance and metabolic adaptation. Increased Wnt activity has been observed in treatment-resistant side populations of CRC cells, while inhibition of Wnt signalling reduces their self-renewal and chemoresistant properties [66]. SEC62 and miR-103/107 have also been shown to maintain stemness-associated and chemoresistant phenotypes through activation or prolongation of Wnt/β-catenin signalling [67,68]. These findings link Wnt activity with the shared treatment-surviving phenotypes described in Section 3.4 and Section 5.4.

Wnt/β-catenin signalling can also influence drug exposure through regulation of ABC transporters. Active β-catenin increases ABCB1 expression and has been associated with other multidrug-efflux mechanisms, thereby reducing intracellular retention of cytotoxic agents [69,70]. Wnt/β-catenin signalling also intersects with anti-apoptotic regulation. *BCL2L2*, which encodes BCL-W, is a direct β-catenin/TCF7L2 transcriptional target in CRC [71]. However, direct functional evidence linking this Wnt–BCL-W relationship to resistance to 5-FU, oxaliplatin or irinotecan has not been established. Wnt signalling can additionally promote metabolic reprogramming towards glycolysis and biosynthesis [72]. In 5-FU-resistant CRC, TRPC5-associated Wnt/β-catenin signalling has been linked to glucose transporter 1 (GLUT1)-associated metabolic adaptation [73], supporting a role for Wnt-associated metabolic reprogramming in treatment resistance.

### 4.3. Drug-Specific Links Between Wnt/β-Catenin Signalling and Chemoresistance

These broader mechanisms also contribute differently to resistance against individual CRC chemotherapies. Long-term 5-FU exposure has been associated with increased Wnt/β-catenin activity in treatment-surviving CRC populations, together with enhanced clonogenic survival and recovery following treatment [74,75,76,77]. More specifically, in 5-FU-resistant HCT-8R cells, Wnt activation suppressed CHK1 and phospho-CHK1, thereby reducing CHK1–p53-dependent apoptosis. WNT3A increased 5-FU resistance, whereas Wnt inhibition restored CHK1 signalling and increased 5-FU sensitivity, with supporting evidence from a xenograft model [78]. These findings support Wnt-mediated CHK1 suppression in this model, but do not establish direct Wnt regulation of upstream ATR. Oxaliplatin resistance can involve mechanistically distinct DNA-repair and metabolic adaptations. SOX9 promotes oxaliplatin resistance through MMS22L-dependent DNA double-strand-break repair [79]; however, a direct Wnt/β-catenin–SOX9–MMS22L relationship was not established in that study. In contrast, direct Wnt-specific evidence has been demonstrated for the Wnt/β-catenin–IMPDH2 axis. In oxaliplatin-resistant CRC cells, Wnt/β-catenin signalling was hyperactivated and showed reciprocal positive regulation with IMPDH2, promoting purine-metabolic adaptation and reduced caspase-dependent apoptosis. Wnt-pathway inhibition resensitised resistant cells to oxaliplatin, whereas guanosine monophosphate (GMP) supplementation restored resistance, supporting a causal role for this axis [80]. Irinotecan resistance can involve adaptation to TOP1 inhibition and replication-associated damage together with reprogramming of the cellular stress response. For irinotecan, limited functional evidence supports a direct contribution of Wnt/β-catenin signalling to drug sensitivity. β-Catenin silencing decreases ABCB1/ABCG2 expression and increases irinotecan sensitivity, while inhibition of the Wnt target IGF2BP1 similarly reduces ABCB1/ABCG2 expression and increases irinotecan sensitivity [66,81]. However, the specific mediation of this effect by ABCB1/ABCG2 remains associative. Accordingly, the defined Wnt-associated 5-FU and oxaliplatin mechanisms provide direct causal preclinical evidence, whereas the overall Wnt–irinotecan relationship remains predominantly associative or indirect/shared adaptive-survival. Direct Wnt regulation of TYMS/DPD-mediated 5-FU resistance, glutathione-mediated oxaliplatin detoxification or platinum transport/accumulation, TOP1 alterations, or UGT1A1-mediated SN-38 metabolism is not established.

### 4.4. Therapeutic Targeting of Wnt/β-Catenin Signalling

Several strategies have been developed to target Wnt/β-catenin signalling in CRC, including inhibition of Wnt-ligand secretion and downstream β-catenin-dependent transcription. Table 1 summarises the principal Wnt/β-catenin-associated mechanisms linked to chemoresistance. Porcupine inhibitors block Wnt-ligand palmitoylation and secretion, thereby suppressing ligand-dependent pathway activation. PORCN inhibitors such as WNT974 (LGK974) and ETC-159 were developed to suppress ligand-dependent Wnt signalling. Early clinical and preclinical data show that these drugs can cause pathway inhibition in certain tumours [82,83,84]. In tumours without truncating *APC* mutations, porcupine inhibition could be effective in CRC when pathway activation remains dependent on extracellular Wnt ligands. Preclinical studies in molecularly selected CRC models harbouring *RSPO2*/*RSPO3* fusions or *RNF43*/*ZNRF3* alterations have shown moderate sensitivity. Porcupine inhibition reduced both Wnt signalling and tumour growth in these models [85,86]. As pathway signalling can occur even without ligand involvement, this method may not be as effective in cases of CRC harbouring truncating *APC* mutations. A potentially adverse effect has also been observed in *Apc*-deficient intestinal models. Porcupine inhibition reduced normal *Lgr5*-positive intestinal stem-cell populations and decreased cellular competition within intestinal crypts. This favoured the fixation and expansion of Apc-deficient cells and accelerated adenoma formation. Therefore, although porcupine inhibition successfully suppresses ligand-dependent Wnt signalling, it may inadvertently create a selective environment favouring ligand-independent *Apc*-mutant cells [87]. Porcupine inhibitors also face clinical translation issues due to their on-target toxicity in normal Wnt-dependent tissues, including bone-related adverse effects. As a result, some early-phase porcupine-inhibitor studies were either discontinued or not advanced to later-stage development [88,89]. Another way to target the Wnt/β-catenin signalling pathway is by the direct inhibition of β-catenin. This includes disrupting the interactions between β-catenin and TCF or between β-catenin and CREB-binding protein (CBP). Direct disruption of β-catenin-dependent transcription has shown preclinical activity in CRC models [90,91]. However, translation of this strategy remains limited by pathway redundancy, molecular heterogeneity and on-target effects in normal Wnt-dependent tissues. An additional limitation is that single-node inhibition can be circumvented by pathway redundancy and mutational heterogeneity [92,93]. Wnt-directed treatment should account for the position and dependence of pathway activation. Ligand-dependent tumours may be more suitable for porcupine inhibition, whereas ligand-independent disease may require downstream targeting. For WNT974 specifically, model-based dose selection has been used to inform its translational clinical development [94].

## 5. Notch and Hippo/YAP1 Signalling

### 5.1. Notch Signalling and Chemoresistance

Notch signalling regulates intestinal epithelial differentiation, proliferation and tissue renewal. Sustained Notch activation in CRC can instead maintain proliferative and stem-like cellular states and impair differentiation. Delta-like ligands (DLLs) and Jagged family ligands, including JAG1, are membrane-bound Notch ligands. In canonical Notch signalling, these membrane-bound ligands bind Notch receptors on adjacent cells. Ligand binding induces sequential proteolytic cleavage of the receptor and release of the Notch intracellular domain (NICD), which translocates to the nucleus and regulates Notch-responsive transcriptional programmes. These programmes are linked to self-renewal and defective differentiation [97]. Controlled Notch activation in normal intestinal epithelium supports absorptive differentiation through NICD–RBPJ–HES1-mediated repression of *ATOH1*, whereas sustained Notch activity in CRC can maintain this repression and reduce the expression of differentiation-associated genes such as *MUC2* [98,99]. Sustained Notch activity can therefore impair terminal differentiation and maintain a proliferative, progenitor-like state [98,99]. In chemoresistant CRC, Notch signalling can reinforce the stem-like and epithelial–mesenchymal plasticity described in Section 3.4 and Section 5.4. Endothelial-derived JAG1 can activate Notch signalling in CRC cells and enhance stem-like properties, illustrating how microenvironmental signals may contribute to these adaptive states [100,101]. NOTCH1 activation has also been associated with epithelial–mesenchymal transition (EMT), whereas NOTCH1 inhibition suppresses EMT-associated programmes [102,103]. Even in CRCs containing *APC* mutations, the level of Wnt/β-catenin activity varies between individual cells. This heterogeneity is influenced by cellular differentiation state and signals from the surrounding microenvironment [104]. Wnt-mediated *JAG1* transcription provides one mechanistic connection through which Wnt activity can enhance Notch signalling and support an undifferentiated state [105,106]. Drug-specific functional evidence has been demonstrated for Notch signalling in oxaliplatin-treated CRC models. Inhibition of oxaliplatin-induced NOTCH1 activation enhances chemosensitivity [107], while the KDM6A/B–H3K27me3–NOTCH2 axis has also been implicated in oxaliplatin resistance [108]. Although the HES1 study did not independently establish upstream NOTCH receptor dependence, HES1, a canonical Notch effector, was functionally implicated in 5-FU resistance through EMT- and ABC-transporter-associated mechanisms in CRC models [58].

### 5.2. Hippo/YAP1 Signalling and Chemoresistance

The Hippo signalling pathway is a conserved regulatory network controlling intestinal epithelial growth, homeostasis and regeneration. Within the canonical Hippo kinase cascade, mammalian sterile 20-like kinases 1 and 2 (MST1/2) activate large tumour suppressor kinases 1 and 2 (LATS1/2). LATS1/2 then phosphorylate YAP1 and transcriptional co-activator with PDZ-binding motif (TAZ; encoded by *WWTR1*), promoting their cytoplasmic retention and degradation. When Hippo kinase signalling is attenuated, YAP1/TAZ accumulate in the nucleus and bind to TEA domain transcription factors (TEADs). YAP1/TAZ–TEAD complexes activate transcriptional programmes involved in proliferation, survival and regenerative plasticity. Nuclear YAP1/TAZ activity in CRC is strongly associated with tumour progression and poor therapeutic response [109,110,111].

In chemoresistant CRC, receptor-mediated signals can promote upstream YAP1 activation. In 5-FU-resistant CRC cells, epidermal growth factor receptor (EGFR) signalling helps maintain YAP1 activity, whereas EGFR inhibition promotes YAP1 phosphorylation and degradation and restores 5-FU sensitivity [112]. Oxaliplatin can induce EDN1 expression and β-arrestin1/YAP1 signalling, while functional studies in oxaliplatin-resistant CRC models support the EDN1–β-arrestin1–YAP1 axis as a mediator of oxaliplatin resistance [113]. A recent CRC study demonstrated that YAP1 promotes 5-FU resistance through the mechanistic target of rapamycin (mTOR)/glucose transporter 3 (GLUT3) axis [114]. In 2026, preferential upregulation of the angiomotin p80 isoform (AMOT-p80) was identified in 5-FU- and oxaliplatin-resistant CRC cells. AMOT depletion increased drug sensitivity and apoptosis while reducing nuclear YAP1. AMOT-p80 re-expression restored nuclear YAP1 and reduced drug sensitivity. These cell-line findings nominate AMOT-p80 as a candidate upstream dependency of YAP1-associated chemoresistance that requires validation in additional models [115].

More broadly, YAP1/TAZ signalling has been implicated in damage tolerance and apoptosis avoidance across treatment-resistant cancers [116,117]. In CRC, YAP1-dependent regenerative plasticity may also contribute to treatment-surviving states, including regeneration-like and fetal-like programmes, although these broader adaptive effects are considered in greater detail in Section 5.4 [109,111,118]. Evidence from other tumour types provides additional proof of principle for targeting this dependency, with YAP1/TEAD inhibition restoring paclitaxel sensitivity in lung cancer models [119] and improving responses to KRASG12C inhibition in other tumour models [120]. Accordingly, the YAP1–mTOR–GLUT3 and EDN1–β-arrestin1–YAP1 axes are classified as direct causal preclinical evidence, whereas c-Yes/YAP1-associated quiescence and regenerative plasticity are classified as indirect/shared adaptive-survival evidence. The drug-specific Hippo/YAP1 relationships and their mechanistic directness and evidence strength are summarised in Table 2.

### 5.3. Crosstalk Between Wnt, Notch and Hippo/YAP1 Signalling

Mechanistic interactions between Wnt/β-catenin, Notch and Hippo/YAP1 signalling can coordinate cellular responses in CRC. Wnt signalling can enhance Notch activity through JAG1 [105,106,122], while Wnt/β-catenin signalling can directly regulate YAP1 expression in CRC cells, providing a mechanistic connection between these pathways [123]. Regeneration- and lineage-plasticity-associated programmes have also been implicated in adaptive cellular responses [124,125,126,127]. More broadly, Wnt signalling contributes to stem-cell maintenance, and β-catenin-dependent transcriptional programmes have been characterised in CRC cells [128,129]. A more directly defined drug-specific interaction involves the ADAM10–NOTCH2–TCF7L2 axis, which links Notch signalling with Wnt transcription in resistant CRC. Functional disruption of this axis reduced Wnt target-gene expression and improved FOLFOX sensitivity, including in resistant organoid models [121].

### 5.4. Therapy-Induced Signalling Adaptation, Cellular Plasticity and Drug-Tolerant Persister States

**Figure 4:**
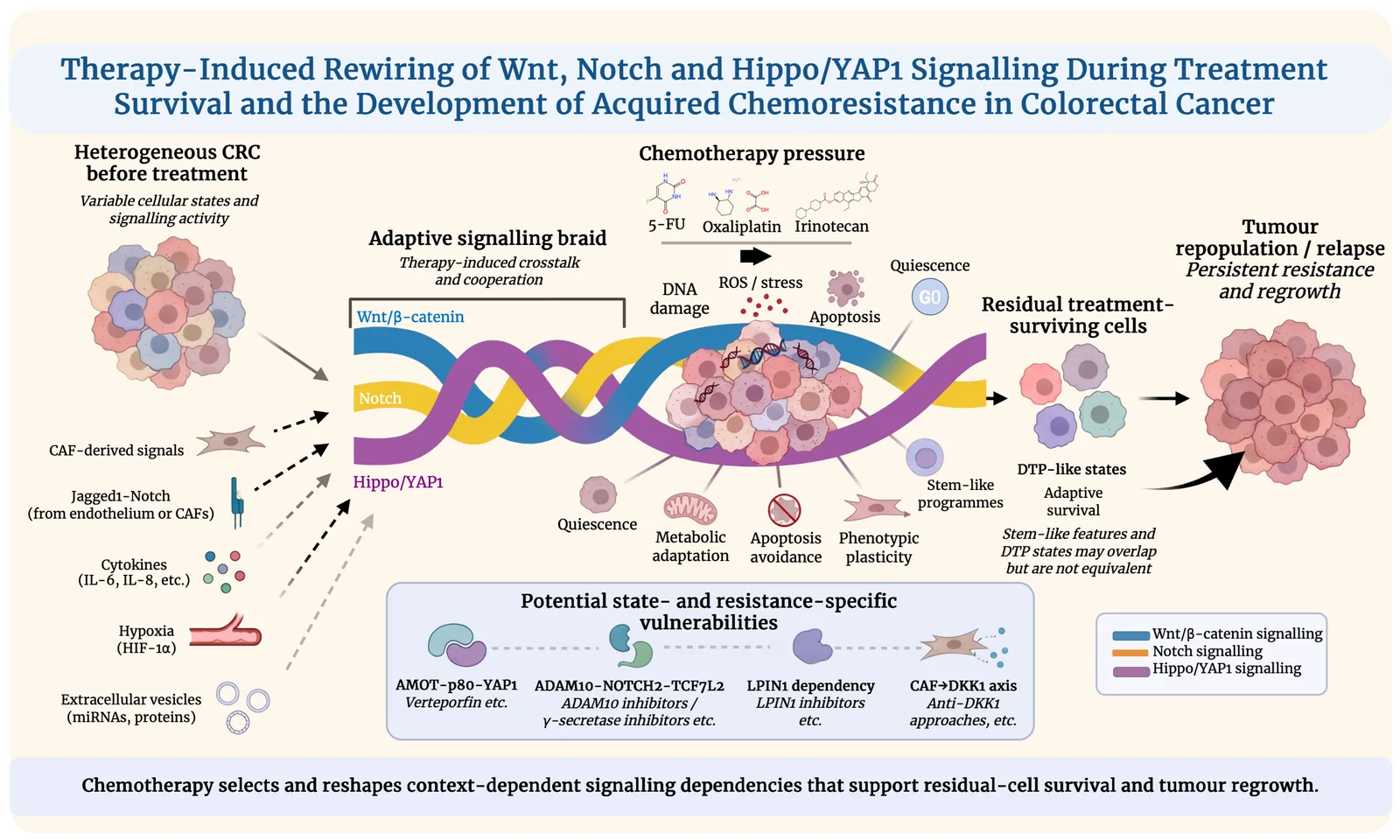
Therapy-induced rewiring of Wnt/β-catenin, Notch and Hippo/YAP1 signalling during treatment survival and the development of acquired colorectal cancer chemoresistance, promoting residual-cell survival, adaptive plasticity and tumour regrowth while revealing state-specific therapeutic vulnerabilities. Created in BioRender. Wilk, I. (2026) https://BioRender.com/j2zwimb.

Chemotherapy can induce reversible DTP states in patient-derived CRC models while preserving clonal diversity. These populations are commonly slow-cycling and may display phenotypic plasticity (Table 3, Figure 4) [130,131,132,133]. Following treatment withdrawal, surviving DTP cells can resume proliferation, as illustrated by MEX3A-positive persister populations that regenerate CRC organoids after treatment [134]. In a separate targeted-therapy context, CRC cells adopted an SMAD1–FGFR3-regulated Paneth-like state during combined KRAS and EGFR inhibition, which promoted treatment resistance [135].

The Hippo/YAP1 signalling pathway can contribute to treatment-surviving cellular states. Upon re-exposure to 5-FU, acquired 5-FU-resistant CRC cells can avoid cell death by entering a reversible quiescent state associated with the c-Yes/YAP1 axis [76]. This state is accompanied by increased expression of stemness-associated genes, including LGR5, ALDH1A3 and POU5F1 (OCT4) [76,136].

Metabolic adaptation can also create targetable dependencies in DTP cells. A 2025 preclinical study found LPIN1 to be upregulated in CRC DTPs, supporting lipid-droplet formation. LPIN1 inhibition or statin treatment disrupted lipid homeostasis and induced ferroptotic cell death in DTPs and patient-derived organoids [137]. These findings identify LPIN1-dependent lipid metabolism as a preclinical vulnerability in CRC DTP populations.

Treatment-associated changes in developmental transcriptional programmes have also been reported. During combined KRASG12C and EGFR inhibition, YAP1 and fetal intestinal signatures were found alongside acquired genetic resistance alterations [138]. Analysis of patient-matched biopsies showed that these genetic alterations were frequently subclonal and were not detected in every patient [138]. In the chemotherapy-specific setting, FOLFIRI exposure increased oncofetal gene expression in patient-derived CRC organoids, while YAP1/activator protein 1 (AP-1) signalling was implicated in maintenance of the oncofetal programme [139].

## 6. Tumour Microenvironment and Translational Applications

### 6.1. Tumour Microenvironment-Mediated Chemoresistance

The tumour microenvironment (TME) provides an important non-cell-autonomous component of acquired chemoresistance in CRC. Cancer-associated fibroblasts (CAFs) can reduce chemotherapy sensitivity through paracrine signalling, extracellular vesicle transfer and stromal remodelling [140,141]. Long-term platinum accumulation within CAFs may further influence local drug handling and contribute to stromal-mediated resistance [142]. Immune and metabolic components of the TME can also support treatment survival. Experimental evidence from carboplatin-treated colorectal cancer cells suggests that IL-6/STAT3 signalling can influence platinum sensitivity, although its applicability to acquired oxaliplatin resistance remains to be established [143]. Separately, colorectal cancer-associated fibroblasts can suppress effector T-cell activity through NECTIN2-dependent interactions [144]. Hypoxia and metabolic stress provide additional selective pressures, with hypoxia-inducible factor 1 alpha (HIF-1α)-associated signalling and transforming growth factor beta (TGF-β)/GLI2- and HIF-1α/miR-338-5p/IL-6-dependent programmes implicated in treatment resistance and survival-associated phenotypes [145,146].

### 6.2. Non-Cell-Autonomous Resistance and Treatment-Induced TME Remodelling

Non-cell-autonomous mechanisms can support treatment survival or reduce chemotherapy sensitivity through signals from nearby stromal and immune components, rather than solely through tumour-cell-intrinsic mechanisms [147]. Cytokine networks are a key part of this process. A 2025 study linked chemotherapy-induced DKK1 secretion by CAFs with CRC chemoresistance and an immunosuppressive TME. DKK1 neutralisation improved 5-FU efficacy in vivo, supporting it as a potential microenvironmental resistance target [148]. Stromal programmes that are activated by TGF-β can also lead to the secretion of interleukin-11 (IL-11). Following its secretion, IL-11 triggers glycoprotein 130 (GP130)/STAT3 signalling. This signalling axis supports metastatic fitness and metastasis initiation [149]. These findings illustrate broader survival effects of stromal cytokine signalling, although the evidence is more directly related to metastatic behaviour. Extracellular vesicles can also promote non-cell-autonomous resistance. Exosomes derived from fibroblasts can prime CRC stem-like cells and enhance their chemoresistance [150]. CAFs release exosomal long non-coding ribonucleic acids (lncRNAs), like *H19* and CRC-associated long non-coding RNA (*CCAL*). Both of these lncRNAs increase oxaliplatin resistance and stem-like properties, whilst activating survival programmes associated with β-catenin [140,151]. Oxaliplatin-resistant tumour cells can also release exosomal miR-208b. These exosomes support the expansion of regulatory T cells (Tregs), which help the CRC cells avoid immune attack [152]. miR-208b promotes Treg expansion and thereby contributes to tumour progression and oxaliplatin resistance through the suppression of PDCD4 in CD4+ T cells [152]. In addition, chemotherapy itself can stimulate CAFs to secrete glutamic acid-leucine-arginine-positive (ELR+) chemokines. Such chemokines can maintain cells with tumour-initiating capacity. This microenvironmental signalling can further support chemoresistance and residual tumour-cell survival after treatment [147]. Other vascular and immune populations may also support or restrict treatment survival according to their functional state [153,154]. By contrast, cytotoxic T cells and natural killer cells may contribute to the elimination of tumour cells. Residual tumour cells may be protected by regulatory T cells and immunosuppressive myeloid populations. The inflammatory and immune organisation within the CRC TME can be further influenced by neutrophils, dendritic cells, B cells and plasma cells [153,154]. Beyond these signalling mechanisms, the TME can also be remodelled by chemotherapy at a cellular and activation state level. Single-cell and spatial profiling of colorectal liver metastases has shown substantial chemotherapy-associated remodelling of the immune microenvironment, including changes in macrophage states and antitumour immune balance [155]. Significant remodelling of CAF subsets was likewise shown in paired pre- and post-treatment rectal cancer samples. Immune activation and tumour suppression were supported by some post-treatment CAF states, while EMT was promoted by fibroblast activation protein-positive CAFs [156].

### 6.3. Biomarkers of Residual Disease, Resistance Mechanisms and Treatment Response

Residual-disease biomarkers identify persistent or evolving disease, whereas mechanism-specific biomarkers may help define the process responsible for resistance. Circulating tumour DNA (ctDNA) is an especially useful indicator. Postoperative or post-treatment ctDNA positivity provides evidence of molecular residual disease and is associated with an increased risk of recurrence [157,158]. Serial ctDNA analysis can support longitudinal monitoring of disease burden and recurrence, but ctDNA positivity alone does not identify the molecular mechanism of chemoresistance; resistance-specific clonal evolution requires variant-level longitudinal analysis. Drug-proximal features involving TYMS/DPD, ERCC1, platinum transport/accumulation, TOP1 or ABC-transporter activity may provide more mechanism-specific information on treatment response, although their clinical validation remains variable [20,21,31,38,43,46]. Functional DNA-repair status may provide more mechanism-specific prediction of chemotherapy response. A 2026 study linked low RAD51 expression and associated homologous-recombination deficiency with FOLFIRI sensitivity using patient-derived xenograft (PDX), tumoroid and clinical-sample analyses [159]. Although the predictive association was validated across complementary models and patient samples within this study, independent prospective validation remains necessary before clinical implementation. Transcriptomic and pathway-state markers may identify broader resistance-associated cellular states, aside from these drug-proximal markers. For example, the consensus molecular subtype (CMS) system identifies a CMS4 subgroup characterised by prominent stromal and mesenchymal features. CMS4 is associated with adverse prognosis, while associations with treatment response are context-dependent and require prospective validation [160,161]. Pathway-state markers may provide additional information on resistance-associated cellular states. Nuclear β-catenin has been associated with resistance to PI3K/AKT-pathway inhibition in CRC [162], whereas YAP1 activity has been linked with chemotherapy resistance and treatment-surviving states [76,163]. These pathway-state markers remain investigational rather than clinically validated predictors of acquired chemoresistance. MEX3A represents a more experimentally defined marker of a chemotherapy-surviving DTP population, although its current evidence remains preclinical [134]. By testing drug responses directly rather than inferring resistance from molecular markers only, patient-derived organoids offer a complementary functional approach. Although broader prospective clinical validation will still be required, their ability to predict CRC treatment response is promising [164].

### 6.4. Molecular Vulnerabilities and Mechanism-Guided Strategies to Overcome Chemoresistance

The strategies discussed below should be distinguished according to both resistance state and translational maturity. Preclinically validated resistance-reversal strategies were defined as interventions in which functional target perturbation restored chemotherapy sensitivity in established resistant CRC models. Interventions that eliminated DTP or other treatment-tolerant populations were classified separately as tolerance-directed strategies. Both provide mechanistic proof-of-concept but should not be interpreted as clinical validation. Strategies supported primarily by mechanistic associations without direct functional reversal or elimination of treatment-tolerant populations are considered therapeutic hypotheses, whereas clinical evidence is discussed separately for interventions evaluated in patients with CRC.

In resistant CRC models, inhibition of EGFR/YAP1 signalling restored 5-FU sensitivity, providing preclinical proof-of-concept for resistance reversal rather than evidence of clinical efficacy [112]. As Wnt/β-catenin, Notch and Hippo/YAP1 signalling also regulate normal intestinal renewal, differentiation and tissue repair, resistance-directed inhibition must achieve sufficient tumour selectivity to avoid unacceptable on-target toxicity [92,165].

Recent 2025–2026 findings involving AMOT-p80–YAP1 and ADAM10–NOTCH2–TCF7L2 identify pathway-specific resistance dependencies; however, these remain preclinical therapeutic vulnerabilities without prospective clinical validation in CRC [115,121]. A tumour-microenvironment-responsive hydrogel delivering *NOTCH1* short-hairpin RNA also suppressed CRC xenograft growth. No significant effects on Notch signalling were observed in adjacent normal tissue [166]. This provides proof-of-concept that spatially selective delivery may reduce the toxicity associated with systemic pathway inhibition. Furthermore, proof of principle for direct YAP1–TEAD inhibition was established by the first-in-human phase I/II trial of the TEAD inhibitor, VT3989. However, responses were mainly seen in mesothelioma rather than CRC [167].

Another resistance-directed approach is provided by DNA-repair dependencies. Targeting LIG1 with gambogenic acid suppressed DNA repair and improved oxaliplatin sensitivity in CRC cells, DTPs, organoids and in vivo models, with combined treatment promoting ZBP1-dependent PANoptosis [34]. ATM inhibition also increased chemotherapy sensitivity in CRC tumoroids, suggesting that DNA-repair states may reveal targetable vulnerabilities [159].

Treatment-surviving states may also expose vulnerabilities that can be targeted before stable acquired resistance develops. LPIN1 inhibition or statin treatment induced ferroptotic death in CRC DTPs and patient-derived organoids, supporting preclinical proof-of-concept for eliminating treatment-tolerant populations rather than clinically validated reversal of acquired resistance [137]. Bone morphogenetic protein 4 (BMP4) can cause CRC cells with stem-like properties to differentiate and lose these features. BMP4-induced differentiation subsequently increased drug sensitivity [168]. Overall, the EHMT2–PPM1B, LIG1, FBXW7–MCL1, AMOT-p80–YAP1 and LPIN1-dependent vulnerabilities discussed in this review have varying degrees of functional preclinical support, but none should currently be interpreted as a clinically validated resistance-reversal or tolerance-directed strategy. Their therapeutic relevance in patients remains to be established prospectively.

More selective preclinical approaches have also emerged. In 2025, KLF5-associated PI3K/AKT activation was identified in oxaliplatin-resistant CRC populations. PI3K inhibition enhanced oxaliplatin efficacy in preclinical models, supporting this axis as another candidate resistance target [169]. Petosemtamab (MCLA-158), a bispecific antibody targeting EGFR and LGR5, was identified through functional screening of tumour-derived and healthy colorectal organoids. In preclinical models, petosemtamab promoted degradation of EGFR in LGR5-positive CRC cells. It also inhibited tumour growth and metastatic initiation. Limited toxicity towards healthy LGR5-positive colonic stem cells was seen with its use [170].

Combination immunotherapy strategies have also been evaluated in microsatellite-stable (MSS) CRC [171,172]. To date, the molecular vulnerabilities summarised in Table 4 have not been prospectively validated as resistance-reversal or tolerance-directed targets in CRC patients. By contrast, several broader combination strategies have reached clinical evaluation, although these trials do not validate the specific resistance dependencies described above. In the phase III CanStem303C trial, adding napabucasin to FOLFIRI did not improve median overall survival in previously treated metastatic CRC, either in the overall population (14.3 versus 13.8 months) or in the phosphorylated STAT3 (pSTAT3)-positive subgroup (13.2 versus 12.1 months) [173]. In the smaller phase II AtezoTRIBE study, first-line folinic acid, 5-fluorouracil, oxaliplatin and irinotecan (FOLFOXIRI)/bevacizumab with or without atezolizumab was evaluated. In its four-year analysis, median overall survival was 33.0 versus 27.2 months (hazard ratio 0.78, 80% confidence interval 0.61–0.98; *p* = 0.084) [174]. As overall survival was not the primary endpoint of this phase II study, this finding should be regarded as preliminary rather than definitive evidence of an overall survival benefit. Exploratory analyses suggested greater benefit in selected proficient mismatch repair (pMMR) tumours with high tumour mutational burden and/or high Immunoscore Immune-Checkpoint status [174]. Given the substantial differences in trial phase, sample size, treatment setting and statistical design, these findings should not be interpreted as equivalent or directly opposing evidence. Table 4 summarises the principal emerging molecular vulnerabilities and mechanism-guided strategies discussed in this review.

### 6.5. Emerging Technologies for Resistance Profiling and Tumour Heterogeneity

Single-cell and spatial profiling can resolve heterogeneous treatment-surviving populations and track changes in their molecular states and interactions during therapy. Single-cell sequencing can identify rare epithelial, stromal and immune subpopulations with DTP-associated transcriptional states, stemness-associated programmes or other plastic phenotypes [175,176]. Single-cell profiling can resolve epithelial tumour states that are obscured by bulk transcriptomic classifications and can refine molecular classification in CRC [177]. When longitudinal samples are available, these approaches may also distinguish resistant populations that were already present before treatment from cellular states that emerge or expand during therapy. Single-cell analysis recently identified an EpC2 epithelial subpopulation associated with oxaliplatin resistance in CRC. Pseudotemporal and cell–cell communication analyses were used to characterise resistance-associated cellular states. Connectivity Map analysis identified dasatinib as a potential therapeutic candidate [178]. Single-cell RNA sequencing was combined with machine learning to identify predicted DTP populations in patient-derived colorectal organoids in a separate 2025 study. In malignant organoids, YM-155 and THZ2 subsequently showed synergistic effects with trametinib [179]. Spatial transcriptomics preserves tissue architecture and can map tumour–stromal and tumour–immune relationships in CRC [180,181]. Integration of spatial transcriptomics with single-cell RNA sequencing has also been directly demonstrated in CRC [182]. As spatial information can determine whether treatment-surviving tumour populations occupy niches associated with CAF signalling, immune suppression, hypoxia or other forms of microenvironment-mediated protection, it is particularly relevant to resistance.

## 7. Discussion

**Figure 5:**
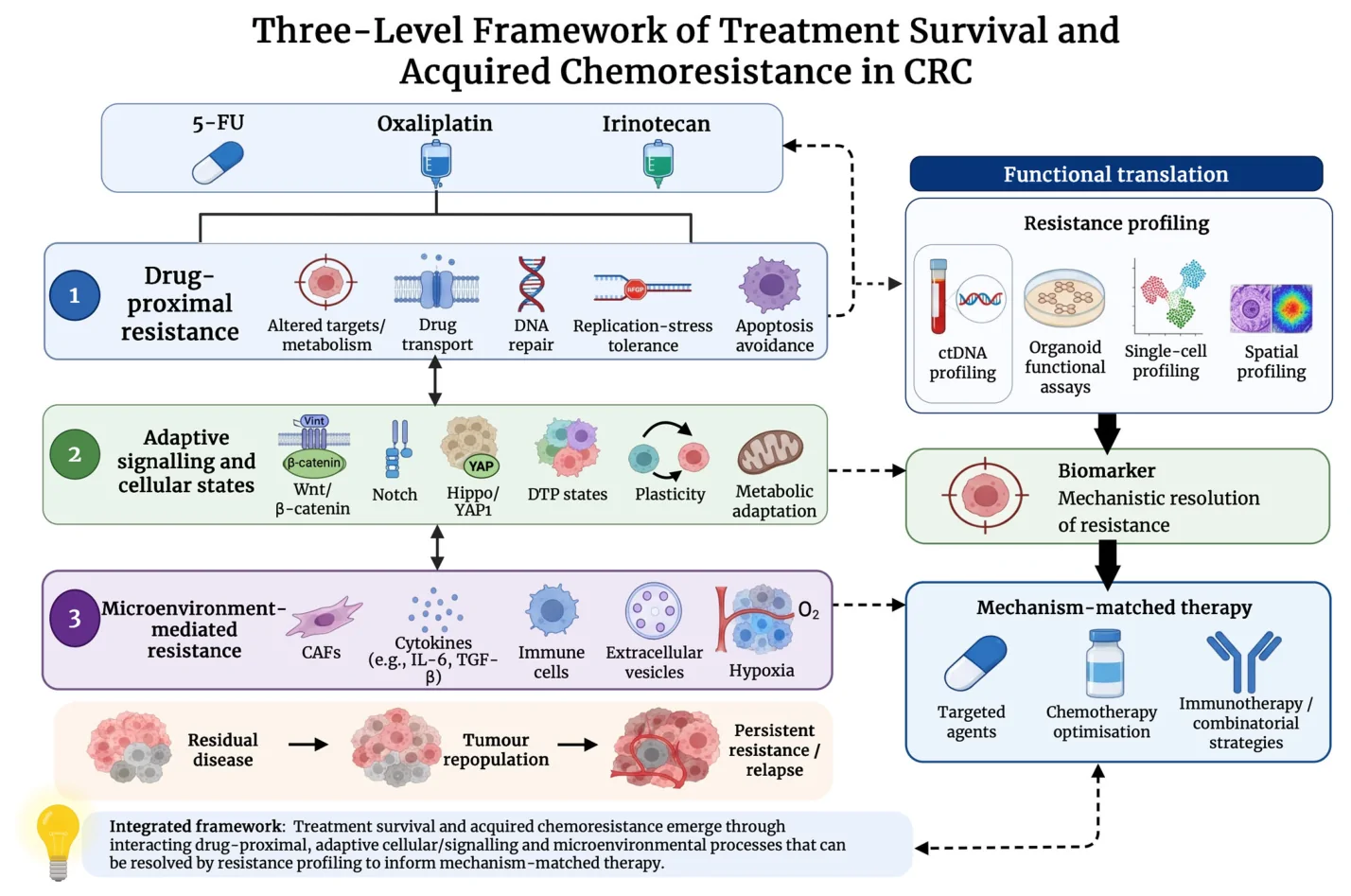
Three-level framework of treatment survival and acquired chemoresistance in colorectal cancer. Drug-proximal resistance, adaptive cellular states and microenvironment-mediated resistance interact to promote tumour persistence and relapse. Resistance profiling may help identify dominant mechanisms and guide mechanism-matched therapy. Created in BioRender. Wilk, I. (2026) https://BioRender.com/ud314d8.

The evidence synthesised in this review supports a three-level framework of processes contributing to treatment survival and acquired chemoresistance in CRC: drug-proximal resistance, adaptive cellular and signalling states, and non-cell-autonomous microenvironment-mediated resistance (Figure 5). These mechanisms are distinct, but they can interact and change under therapeutic pressure. Importantly, this framework extends beyond pathway-level description by evaluating resistance mechanisms within the context of the individual chemotherapeutic agent and according to the strength and mechanistic directness of the supporting evidence. Drug-proximal resistance directly alters the interaction between the tumour cell and the chemotherapeutic agent at the first level. Resistance to 5-FU, oxaliplatin and irinotecan can arise through different drug-proximal mechanisms. These include altering drug metabolism or transport, modifying targets, enhancing DNA repair and replication-stress tolerance, and the avoidance of apoptosis [16,20,31,43].

Beyond these drug-specific mechanisms, Wnt/β-catenin, Notch and Hippo/YAP1 signalling are also recurrently implicated in CRC chemoresistance. These pathways can be considered part of a second level of adaptive cellular and signalling states, although defined drug-specific pathway dependencies are also supported by direct evidence. Their roles are treatment- and mechanism-dependent rather than universal, with stronger direct evidence for specific 5-FU-, oxaliplatin- and FOLFOX-associated mechanisms than for irinotecan. Evidence of interactions between these signalling pathways, including the functionally characterised ADAM10–NOTCH2–TCF7L2 axis in FOLFOX-resistant models, supports cooperative signalling in defined treatment contexts [121]. However, pathway crosstalk does not independently establish direct regulation of other drug-proximal resistance mechanisms. Links to TYMS/DPD regulation, glutathione-mediated oxaliplatin detoxification or platinum transport/accumulation, and TOP1 expression changes or *TOP1* mutations have not been established. Accordingly, pathway activation associated with treatment survival should not automatically be interpreted as the direct molecular cause of every form of chemoresistance. Adaptive survival also includes reversible DTP and other plastic cellular states. These states may overlap with stem-like programmes but should not be treated as equivalent to stable acquired resistance or functional CSC identity [131,132,133,134,183]. Reversible survival during treatment, persistent resistance after drug withdrawal and rechallenge, and functional self-renewal or tumour-initiating capacity represent distinct biological properties. Maintaining these distinctions therefore helps avoid attributing stable chemoresistance to transient treatment-surviving populations.

The TME adds a further non-cell-autonomous component to resistance. Stromal, immune, vascular and metabolic components can alter chemotherapy response through interactions that protect tumour cells or modify the selective pressures imposed by treatment. The cellular composition and activation states of the TME can also be remodelled by chemotherapy itself. This indicates that acquired chemoresistance is both spatially and temporally heterogeneous, and that the dominant mechanism may differ between cellular populations, anatomical niches and treatment stages. Compared with recent broad reviews of CRC chemoresistance, the present synthesis evaluates pathway involvement within specific drug and resistance contexts. It also distinguishes direct drug-proximal resistance from broader adaptive cellular and microenvironmental processes according to the strength and directness of the supporting evidence [17,18].

The dominant mechanism or cellular state underlying treatment failure is also likely to influence the appropriate biomarker and therapeutic target. Biomarkers of altered targets, drug metabolism, transport or DNA repair may be required for drug-proximal resistance. Adaptive cellular and signalling states may be better characterised by pathway activity, DTP-associated dependencies or functional testing in patient-derived organoids. Stromal, immune or spatial biomarkers may be more informative where non-cell-autonomous resistance dominates. Serial ctDNA can monitor residual or evolving disease, whereas patient-derived organoids, single-cell sequencing and spatial transcriptomics may help define the resistance process and when it emerges [157,158,164,175,180]. Clinical responses to combination strategies in metastatic CRC remain heterogeneous and appear to depend on treatment context and tumour biology. The available findings also suggest that biomarker-based patient selection may be important for identifying subgroups most likely to benefit from treatment intensification [173,174].

Much of the mechanistic evidence remains preclinical, and pathway-specific evidence is stronger for 5-FU and oxaliplatin than for irinotecan. In particular, EHMT2–PPM1B, LIG1 and FBXW7–MCL1 represent functionally supported preclinical resistance-reversal vulnerabilities, whereas AMOT-p80–YAP1 represents an earlier-stage preclinical resistance-associated vulnerability and LPIN1-dependent DTP metabolism represents a tolerance-directed vulnerability [27,34,51,115,137]. None of these vulnerabilities has been clinically validated in CRC (Table 4). The structured evidence appraisal further highlights substantial variation in the strength and translational maturity of the evidence synthesised in this review. Direct functional evidence for several resistance mechanisms has been reproduced across complementary CRC models, including organoid and in vivo systems, supporting moderate evidence for these relationships. However, other proposed mechanisms remain supported predominantly by cell-line or single-model studies and were therefore classified as limited evidence. Strong evidence was less common and was restricted to relationships supported by concordant mechanistic findings together with human or clinical validation. Importantly, pathway–drug relationships were stratified according to mechanistic directness as direct causal, associative, indirect/shared adaptive-survival, or not established, separately from the overall evidence-strength grade. Mechanistic plausibility should likewise not be considered equivalent to clinical validation, and findings reported in a single recent study were regarded as emerging rather than established evidence, irrespective of the breadth of experimental models used within that study. Taken together, this evidence-weighted stratification distinguishes mechanisms directly implicated in drug-specific resistance from broader adaptive or microenvironmental processes that may support treatment survival.

### Limitations of the Review

Several limitations of this review should be acknowledged. First, the literature search was restricted to PubMed and PubMed Central, and relevant studies indexed exclusively in other databases or available through grey literature may therefore have been missed. In addition, 7 of the 248 reports sought for full-text retrieval could not be obtained. Although these represented a small proportion of the reports sought (2.8%), their eligibility and potential contribution to the synthesis could not be assessed, and a minor risk of retrieval bias therefore cannot be excluded. Second, substantial heterogeneity existed among the experimental resistance models included in the literature. Studies differed in cell lines and model systems, drug concentrations, duration and pattern of drug exposure, selection protocols, treatment-free intervals and criteria used to define chemoresistance. Consequently, acute treatment tolerance, persistent drug-tolerant states and stable acquired resistance were not uniformly distinguished across the original studies. Although these phenotypes were differentiated during the present synthesis, variation in the underlying experimental definitions limits direct comparison between studies.

Primary studies and review articles were also included within the screened evidence base. Review articles were used principally to provide mechanistic, methodological or clinical context and were not independently used to increase evidence-strength grades. Nevertheless, their inclusion may introduce some overlap in the evidence discussed. In addition, publication bias cannot be excluded, as positive or mechanistically significant resistance findings may be more likely to be reported than negative or non-confirmatory results. Quantitative synthesis was not performed because of substantial heterogeneity in experimental models, interventions, resistance definitions and reported outcomes. Finally, much of the available evidence remains preclinical, with many proposed mechanisms supported predominantly by cell-line, organoid or animal studies and comparatively limited validation in patient samples or prospective clinical studies. These limitations should be considered when interpreting the translational significance of the mechanisms and therapeutic strategies discussed in this review.

## 8. Conclusions

The success of CRC treatment remains significantly limited by acquired chemoresistance, which is mechanistically heterogeneous and reflects interactions between drug-specific resistance mechanisms, adaptive cellular states and the tumour microenvironment. The available evidence indicates that these processes should be distinguished according to their mechanistic directness, resistance context and level of experimental support rather than interpreted as equivalent contributors to treatment failure.

Important evidence gaps remain, as many proposed resistance dependencies are supported predominantly by preclinical models, with limited prospective validation in patient populations and weaker direct pathway-specific evidence in some treatment contexts, particularly for irinotecan. Future studies should prioritise prospective validation of mechanism-specific biomarkers and resistance-directed therapeutic strategies, supported by longitudinal molecular profiling, ctDNA assessment and patient-derived functional models. Biomarker-selected and mechanism-matched approaches may ultimately provide a more rational basis for preventing stable acquired resistance, treatment failure and relapse.

## Figures and Tables

**Table 1:** Experimentally supported Wnt/β-catenin-associated mechanisms of chemotherapy resistance and treatment survival in colorectal cancer.

Mechanistic Category	Wnt/β-Catenin-Associated Molecular Axis	Experimentally Demonstrated Resistance-Associated Effect	Treatment Context	Evidence Category
Residual stem-like cell survival and tumour repopulation	5-FU-induced p53–WNT3–β-catenin signalling [95]	Activates Wnt/β-catenin signalling and enriches residual stem-like cells; Wnt inhibition suppresses their enrichment and post-treatment tumour regrowth.	5-FU	In vitro, organoid/PDO, in vivo
ABC-transporter-associated chemoresistance	IGF2BP1-associated ABCB1/ABCG2 regulation in Wnt-active CRC cells [81]	IGF2BP1 inhibition reduces *ABCB1/ABCG2* mRNA expression and increases sensitivity to 5-FU, oxaliplatin and irinotecan; transporter mediation of chemosensitisation was not directly demonstrated.	5-FU, oxaliplatin and irinotecan	In vitro
Glycolytic metabolic adaptation	TRPC5–Wnt/β-catenin-associated GLUT1 signalling [73,96]	TRPC5 and GLUT1 expression are increased in 5-FU-resistant CRC cells; TRPC5 knockdown and Wnt/β-catenin inhibition reduce GLUT1 expression, while high TRPC5/GLUT1 expression is associated with 5-FU chemotherapy failure.	5-FU	In vitro, retrospective clinical
Purine-metabolic adaptation and apoptosis avoidance	Wnt/β-catenin–IMPDH2 axis [80]	Promotes IMPDH2-dependent purine metabolism and suppresses caspase-dependent apoptosis; inhibition of Wnt/β-catenin signalling or IMPDH2 restores oxaliplatin sensitivity.	Oxaliplatin	In vitro, in vivo

Note: Evidence categories indicate the supporting primary study/model type(s) and were standardised as in vitro, organoid/PDO, in vivo, human translational, retrospective clinical, prospective clinical or clinical-trial evidence, as applicable. Multiple categories may apply to a single relationship. References cited with each molecular axis support the corresponding mechanistic effect, treatment context and evidence category presented in that row.

**Table 2:** Mechanistic directness, evidence category and evidence strength of drug-specific resistance and treatment-survival mechanisms linked to Wnt/β-catenin, Notch and Hippo/YAP1 signalling in colorectal cancer.

Drug	Resistance Mechanism or Treatment-Survival State	Pathway Connection	Mechanistic Directness	Evidence Category (Study/Model)	Evidence Grade	Key Resistance Effect
5-FU	Apoptosis avoidance in acquired-resistant cells	Wnt-mediated CHK1 suppression [78]	Direct causal	In vitro, in vivo	Moderate	Suppresses CHK1–p53-dependent apoptosis and increases 5-FU resistance.
5-FU	EMT- and ABC-transporter-associated resistance	HES1, a canonical Notch effector [58]	Direct causal at HES1-effector level	In vitro, retrospective clinical	Limited	HES1 promotes 5-FU resistance with EMT- and ABC-transporter-associated changes; receptor-level Notch dependence was not established
5-FU	Glycolytic metabolic adaptation and apoptosis avoidance	YAP1–mTOR–GLUT3 axis [114]	Direct causal	In vitro, in vivo	Moderate	Promotes glycolytic adaptation, apoptosis avoidance and 5-FU resistance.
5-FU	Reversible quiescent treatment-surviving state in acquired-resistant cells	c-Yes/YAP1-associated quiescence [76]	Indirect/shared adaptive-survival	In vitro, human translational	Limited	Supports reversible quiescent survival during 5-FU re-exposure rather than establishing stable resistance.
5-FU	TYMS overexpression, DPD-mediated catabolism and altered nucleotide metabolism [20,21,22,23,24]	No direct Wnt, Notch or Hippo/YAP1 regulation identified	Not established	In vitro, in vivo, human translational	Not established	Maintains nucleotide availability or reduces active 5-FU exposure; direct pathway-specific involvement is not established.
5-FU/oxaliplatin	YAP1-associated survival in established resistant cells	AMOT-p80–YAP1 [115]	Direct causal	In vitro	Limited	Maintains YAP1-dependent survival and reduced sensitivity to 5-FU and oxaliplatin in resistant-cell models.
Oxaliplatin	Purine-metabolic adaptation and apoptosis avoidance	Wnt/β-catenin–IMPDH2 axis [80]	Direct causal	In vitro, in vivo	Moderate	Promotes IMPDH2-dependent purine metabolism and apoptosis avoidance during oxaliplatin treatment.
Oxaliplatin	Chemotherapy-induced prosurvival signalling	Oxaliplatin-induced NOTCH1 signalling with HES1 activation [107]	Direct causal	In vitro	Limited	NOTCH1 activation promotes prosurvival signalling and reduces oxaliplatin sensitivity; HES1 is activated downstream.
Oxaliplatin	NOTCH2-associated stemness and survival	KDM6A/B–H3K27me3–NOTCH2 axis [108]	Direct causal	In vitro, in vivo, retrospective clinical	Moderate	Promotes NOTCH2-dependent survival and oxaliplatin resistance.
Oxaliplatin	YAP1-dependent survival and apoptosis avoidance	EDN1–β-arrestin1–YAP1 axis [113]	Direct causal	In vitro, in vivo	Moderate	Promotes YAP1-dependent survival, apoptosis avoidance and oxaliplatin resistance.
Oxaliplatin	Glutathione-mediated detoxification and altered platinum transport/accumulation [35,36,37,38,39]	No direct Wnt, Notch or Hippo/YAP1 regulation identified	Not established	In vitro, in vivo, retrospective clinical	Not established	Enhances platinum detoxification or alters intracellular drug accumulation; reduced CTR1-mediated resistance is not established, and direct pathway-specific involvement has not been demonstrated.
FOLFOX (5-FU + oxaliplatin)	Adaptive pathway-dependent resistance	ADAM10–NOTCH2–TCF7L2-mediated Wnt transcription [121]	Direct causal	Organoid/PDO, in vivo	Moderate	Maintains Wnt transcription and reduces FOLFOX sensitivity.
Irinotecan	ABC-transporter-associated multidrug resistance	Wnt/β-catenin- and IGF2BP1-associated ABCB1/ABCG2 regulation [66,81]	Direct causal for pathway–drug sensitivity; transporter mediation associative	In vitro	Limited	Wnt/β-catenin or IGF2BP1 inhibition reduces ABCB1/ABCG2 expression and increases irinotecan sensitivity; mediation of chemosensitisation by these transporters remains associative.
Irinotecan	TOP1 alterations, including resistance-associated *TOP1* mutations, UGT1A1-mediated SN-38 metabolism and replication-stress tolerance [43,44,45,46,47,48,49,50]	No direct Wnt, Notch or Hippo/YAP1 regulation identified	Not established	In vitro, in vivo, prospective clinical	Not established	TOP1 alterations can reduce SN-38 sensitivity, UGT1A1 influences SN-38 exposure, and replication-stress responses can support irinotecan survival; direct Wnt, Notch or Hippo/YAP1 involvement is not established.

Note: Mechanistic directness and evidence grade were defined according to Section 2.4. Mechanistic directness was standardised as direct causal, associative, indirect/shared adaptive-survival, or not established. Evidence categories were standardised as in vitro, organoid/PDO, in vivo, human translational, retrospective clinical, prospective clinical, or clinical-trial evidence, as applicable. Multiple evidence categories may apply to a single relationship. References cited in each row support the corresponding resistance mechanism or treatment-survival state, key resistance effect, treatment context and evidence category, as applicable. “Not established” indicates that no direct pathway-specific relationship was identified; in these rows, the cited references support the underlying drug-proximal resistance mechanism rather than proving the absence of a Wnt/β-catenin, Notch or Hippo/YAP1 relationship.

**Table 3:** Experimentally Defined Treatment-Surviving Cellular States and Stable Acquired Resistance Phenotypes Relevant to Chemoresistance in Colorectal Cancer.

Treatment-Survival or Resistance Phenotype	Defining Experimental Characteristics	Molecular or Functional Readouts	Treatment Context	Evidence Category (Study/Model)	Reported Outcome
c-Yes/YAP1-associated quiescent state [76]	Acquired 5-FU-resistant CRC cells enter a reversible, slow-cycling G0 state upon renewed 5-FU exposure.	G0 arrest, c-Yes activation and membrane localisation, c-Yes/YAP1 dissociation and depletion of nuclear YAP1.	Re-exposure of acquired 5-FU-resistant CRC cells to 5-FU.	In vitro, human translational	Survival during 5-FU re-exposure through reversible quiescence rather than establishment of a new stable resistant state.
Diapause-like DTP state [132]	CRC cells enter a reversible, slow-cycling drug-tolerant state while retaining clonal diversity.	Reduced proliferation, diapause-like transcriptional and functional features, chemotherapy survival and renewed growth after treatment withdrawal.	Chemotherapy-treated patient-derived CRC models.	In vitro, in vivo	Reversible treatment persistence followed by resumption of tumour growth after chemotherapy cessation.
MEX3A-positive DTP state [134]	A latent MEX3A-positive population survives chemotherapy and subsequently resumes proliferation.	MEX3A expression, persister survival, organoid regeneration and post-treatment clonal expansion.	Chemotherapy-treated patient-derived CRC organoid and in vivo models.	Organoid/PDO, in vivo	Post-chemotherapy tumour regeneration, identifying MEX3A-positive persisters as a candidate cellular origin of relapse.
Stable acquired 5-FU-resistant state [16]	HCT116-derived ContinB and ContinD cells generated by continuous 5-FU exposure exhibit established moderate or strong 5-FU resistance.	Reduced 5-FU-induced apoptosis, altered cell-cycle responses and differential recovery following drug removal.	Established 5-FU-resistant CRC cells challenged with 5-FU and followed during drug-free recovery.	In vitro	Persistent resistance-associated survival, with more rapid recovery of proliferation in the strongly resistant ContinD cells.

Note: Evidence categories indicate the supporting primary study/model type(s) and were standardised as in vitro, organoid/PDO, in vivo, human translational, retrospective clinical, prospective clinical or clinical-trial evidence, as applicable. Multiple categories may apply to a single relationship. References cited with each phenotype support the corresponding defining experimental characteristics, molecular or functional readouts, treatment context, evidence category and reported outcome presented in that row. Phenotype classification follows the operational definitions provided in Section 2.3.

**Table 4:** Emerging Molecular Vulnerabilities and Mechanism-Guided Strategies for Acquired Chemoresistance and Treatment-Tolerant States in Colorectal Cancer.

Treatment-Response Context	Molecular Vulnerability/Target	Candidate Biomarker or Selection Feature	Resistance-Overcoming Strategy	Evidence Category (Study/Model)	Evidence Grade	Translational Status
Acquired 5-FU resistance	EHMT2–PPM1B [27]	Elevated EHMT2 expression in 5-FU-resistant or non-responsive CRC	EHMT2 inhibition to restore 5-FU sensitivity	In vitro, organoid/PDO, in vivo, retrospective clinical	Moderate	Preclinically validated resistance-reversal strategy; no prospective clinical validation in CRC
Oxaliplatin resistance	LIG1-dependent DNA repair [34]	High LIG1 expression associated with reduced oxaliplatin sensitivity	LIG1 targeting with gambogenic acid to enhance oxaliplatin sensitivity	In vitro, organoid/PDO, in vivo	Moderate	Preclinically validated resistance-reversal strategy; no prospective clinical validation in CRC
Irinotecan resistance	FBXW7–MCL1 [51]	FBXW7 deficiency or *FBXW7* mutation with MCL1 stabilisation	MCL1 inhibition with maritoclax to restore irinotecan/SN-38 sensitivity	In vitro, in vivo, human translational	Moderate	Preclinically validated resistance-reversal strategy; no prospective clinical validation in CRC
5-FU/oxaliplatin resistance	AMOT-p80–YAP1 [115]	AMOT-p80 upregulation and nuclear YAP1	AMOT-p80 depletion to increase 5-FU/oxaliplatin sensitivity	In vitro	Limited	Early preclinical resistance-reversal vulnerability demonstrated in resistant-cell models; no clinical validation in CRC
FOLFOX resistance	ADAM10–NOTCH2–TCF7L2 [121]	High ADAM10/NOTCH2 expression with Wnt-pathway activation	Disruption of the ADAM10–NOTCH2–TCF7L2 axis to enhance FOLFOX sensitivity	Organoid/PDO, in vivo	Moderate	Preclinically validated resistance-reversal strategy; no prospective clinical validation in CRC
DTP-associated treatment tolerance	LPIN1-dependent lipid metabolism [137]	LPIN1-high DTP phenotype and increased lipid-droplet accumulation	LPIN1 inhibition or statin treatment to induce ferroptotic death in DTPs	In vitro, organoid/PDO, in vivo	Moderate	Preclinically validated tolerance-directed strategy; no prospective clinical validation in CRC
TME-mediated chemotherapy resistance	CAF-derived DKK1 [148]	Chemotherapy-induced DKK1 expression/secretion by CAFs	DKK1 neutralisation combined with chemotherapy	In vitro, in vivo, human translational	Moderate	Preclinically validated TME-directed resistance strategy; no prospective clinical validation in CRC

Note: Evidence categories indicate the supporting primary study/model type(s) and were standardised as in vitro, organoid/PDO, in vivo, human translational, retrospective clinical, prospective clinical or clinical-trial evidence, as applicable. Multiple categories may apply to a single relationship. References cited with each molecular vulnerability/target support the corresponding candidate biomarker or selection feature, experimentally evaluated resistance-overcoming strategy and supporting study/model category presented in that row. Evidence grades were assigned separately according to the structured framework described in Section 2.4. Translational status reflects the evidence synthesis performed in this review and distinguishes preclinically validated resistance-reversal strategies from tolerance-directed and TME-directed strategies and from prospective clinical validation; functional activity in experimental models should not be interpreted as evidence of clinical efficacy.

## Data Availability

No new data were created or analysed in this study. Data sharing is not applicable to this article.

## Supplementary Materials

The following supporting information can be downloaded at: https://www.mdpi.com/article/10.3390/cells15191776/s1, Table S1. Database-specific search strategies used for literature identification, Table S2. Structured evidence appraisal of all publications included in the review.
