# Supplementary material for: Acquired Chemoresistance in Colorectal Cancer: Therapy-Induced Signalling Adaptation, Cellular Plasticity and Mechanism-Guided Therapeutic Strategies
Source: Cells. 2026 Sep 29;15(19):1776. doi: 10.3390/cells15191776 (PMC13664393; doi:10.3390/cells15191776)
Supplement: Supplementary file 1 [file cells-15-01776-s001.zip › cells-4556787-supplementary.pdf]

Supplementary Table S1 and Supplementary Table S2

Supplementary Table S1: Database-specific search strategies used for literature identification

Searches were last conducted on 20 August 2026. The expanded search strings are reproduced verbatim from the PubMed and PMC Search Details following automatic term mapping and were not manually modified. “AND” was used to combine major search concepts and “OR” to combine synonymous or related terms. Field tags included MeSH Terms, Supplementary Concept and All Fields where applicable. No language, study-design, publication-type or publication-date filters were applied at the database-search stage.

Database	Search concept	Exact search string	Filters/limits
PubMed	colorectal cancer acquired chemoresistance	("colorectal neoplasms"[MeSH Terms] OR ("colorectal"[All Fields] AND "neoplasms"[All Fields]) OR "colorectal neoplasms"[All Fields] OR ("colorectal"[All Fields] AND "cancer"[All Fields]) OR "colorectal cancer"[All Fields]) AND ("acquirable"[All Fields] OR "acquire"[All Fields] OR "acquired"[All Fields] OR "acquirement"[All Fields] OR "acquirements"[All Fields] OR "acquires"[All Fields] OR "acquiring"[All Fields]) AND ("chemoresistance"[All Fields] OR "chemoresistant"[All Fields] OR "chemoresistence"[All Fields] OR "chemoresistent"[All Fields])	None
PubMed	colorectal cancer 5-FU resistance	("colorectal neoplasms"[MeSH Terms] OR ("colorectal"[All Fields] AND "neoplasms"[All Fields]) OR "colorectal neoplasms"[All Fields] OR ("colorectal"[All Fields] AND "cancer"[All Fields]) OR "colorectal cancer"[All Fields]) AND ("fluorouracil"[Supplementary Concept] OR "fluorouracil"[All Fields] OR "5 fu"[All Fields] OR "fluorouracil"[MeSH Terms]) AND ("resist"[All Fields] OR "resistance"[All Fields] OR "resistances"[All Fields] OR "resistant"[All Fields] OR "resistants"[All Fields] OR "resisted"[All Fields] OR "resistence"[All Fields] OR "resistences"[All Fields] OR "resistent"[All Fields] OR "resistibility"[All Fields] OR "resisting"[All Fields] OR "resistive"[All Fields] OR "resistively"[All Fields] OR "resistivities"[All Fields] OR "resistivity"[All Fields] OR "resists"[All Fields])	None
PubMed	colorectal cancer oxaliplatin resistance	("colorectal neoplasms"[MeSH Terms] OR ("colorectal"[All Fields] AND "neoplasms"[All Fields]) OR "colorectal neoplasms"[All Fields] OR ("colorectal"[All Fields] AND "cancer"[All Fields]) OR "colorectal cancer"[All Fields]) AND ("oxaliplatin"[Supplementary Concept] OR "oxaliplatin"[All Fields] OR "oxaliplatin"[MeSH Terms] OR "oxaliplatine"[All Fields] OR "oxaliplatin s"[All Fields]) AND ("resist"[All Fields] OR "resistance"[All Fields] OR "resistances"[All Fields] OR "resistant"[All Fields] OR "resistants"[All Fields] OR "resisted"[All Fields] OR "resistence"[All Fields] OR "resistences"[All Fields] OR "resistent"[All Fields] OR "resistibility"[All Fields] OR "resisting"[All Fields] OR "resistive"[All Fields] OR "resistively"[All Fields] OR "resistivities"[All Fields] OR "resistivity"[All Fields] OR "resists"[All Fields])	None

Supplementary Table S1 and Supplementary Table S2

PubMed	colorectal cancer irinotecan resistance	("colorectal neoplasms"[MeSH Terms] OR ("colorectal"[All Fields] AND "neoplasms"[All Fields]) OR "colorectal neoplasms"[All Fields] OR ("colorectal"[All Fields] AND "cancer"[All Fields]) OR "colorectal cancer"[All Fields]) AND ("irinotecan"[Supplementary Concept] OR "irinotecan"[All Fields] OR "irinotecan"[MeSH Terms] OR "irinotecan s"[All Fields]) AND ("resist"[All Fields] OR "resistance"[All Fields] OR "resistances"[All Fields] OR "resistant"[All Fields] OR "resistants"[All Fields] OR "resisted"[All Fields] OR "resistence"[All Fields] OR "resistences"[All Fields] OR "resistent"[All Fields] OR "resistibility"[All Fields] OR "resisting"[All Fields] OR "resistive"[All Fields] OR "resistively"[All Fields] OR "resistivities"[All Fields] OR "resistivity"[All Fields] OR "resists"[All Fields])	None
PubMed	colorectal cancer Wnt beta-catenin chemoresistance	("colorectal neoplasms"[MeSH Terms] OR ("colorectal"[All Fields] AND "neoplasms"[All Fields]) OR "colorectal neoplasms"[All Fields] OR ("colorectal"[All Fields] AND "cancer"[All Fields]) OR "colorectal cancer"[All Fields]) AND "Wnt"[All Fields] AND ("ctnnb1 protein human"[Supplementary Concept] OR "ctnnb1 protein human"[All Fields] OR "beta catenin"[All Fields] OR "beta catenin"[Supplementary Concept] OR "beta catenin"[MeSH Terms] OR ("beta"[All Fields] AND "catenin"[All Fields])) AND ("chemoresistance"[All Fields] OR "chemoresistant"[All Fields] OR "chemoresistence"[All Fields] OR "chemoresistent"[All Fields])	None
PubMed	colorectal cancer Notch chemoresistance	("colorectal neoplasms"[MeSH Terms] OR ("colorectal"[All Fields] AND "neoplasms"[All Fields]) OR "colorectal neoplasms"[All Fields] OR ("colorectal"[All Fields] AND "cancer"[All Fields]) OR "colorectal cancer"[All Fields]) AND ("notch"[All Fields] OR "notch s"[All Fields] OR "notched"[All Fields] OR "notches"[All Fields] OR "notching"[All Fields] OR "notchings"[All Fields] OR "notchs"[All Fields]) AND ("chemoresistance"[All Fields] OR "chemoresistant"[All Fields] OR "chemoresistence"[All Fields] OR "chemoresistent"[All Fields])	None
PubMed	colorectal cancer YAP chemoresistance	("colorectal neoplasms"[MeSH Terms] OR ("colorectal"[All Fields] AND "neoplasms"[All Fields]) OR "colorectal neoplasms"[All Fields] OR ("colorectal"[All Fields] AND "cancer"[All Fields]) OR "colorectal cancer"[All Fields]) AND "YAP"[All Fields] AND ("chemoresistance"[All Fields] OR "chemoresistant"[All Fields] OR "chemoresistence"[All Fields] OR "chemoresistent"[All Fields])	None
PubMed	colorectal cancer stem cells chemoresistance	("colorectal neoplasms"[MeSH Terms] OR ("colorectal"[All Fields] AND "neoplasms"[All Fields]) OR "colorectal neoplasms"[All Fields] OR ("colorectal"[All Fields] AND "cancer"[All Fields]) OR "colorectal cancer"[All Fields]) AND ("stem cells"[MeSH Terms] OR ("stem"[All Fields] AND "cells"[All Fields]) OR "stem cells"[All Fields]) AND ("chemoresistance"[All Fields] OR "chemoresistant"[All Fields] OR	None

Supplementary Table S1 and Supplementary Table S2

		"chemoresistence"[All Fields] OR "chemoresistent"[All Fields])	
PubMed	colorectal cancer drug tolerant persister plasticity	("colorectal neoplasms"[MeSH Terms] OR ("colorectal"[All Fields] AND "neoplasms"[All Fields]) OR "colorectal neoplasms"[All Fields] OR ("colorectal"[All Fields] AND "cancer"[All Fields]) OR "colorectal cancer"[All Fields]) AND "drug"[All Fields] AND ("immune tolerance"[MeSH Terms] OR ("immune"[All Fields] AND "tolerance"[All Fields]) OR "immune tolerance"[All Fields] OR "tolerance"[All Fields] OR "drug tolerance"[MeSH Terms] OR ("drug"[All Fields] AND "tolerance"[All Fields]) OR "drug tolerance"[All Fields] OR "tolerabilities"[All Fields] OR "tolerability"[All Fields] OR "tolerable"[All Fields] OR "tolerableness"[All Fields] OR "tolerably"[All Fields] OR "tolerances"[All Fields] OR "tolerant"[All Fields] OR "tolerants"[All Fields] OR "tolerate"[All Fields] OR "tolerated"[All Fields] OR "tolerates"[All Fields] OR "tolerating"[All Fields] OR "toleration"[All Fields] OR "tolerator"[All Fields] OR "tolerators"[All Fields] OR "tolerence"[All Fields]) AND ("persist"[All Fields] OR "persistance"[All Fields] OR "persistant"[All Fields] OR "persisted"[All Fields] OR "persistence"[All Fields] OR "persistences"[All Fields] OR "persistencies"[All Fields] OR "persistency"[All Fields] OR "persistent"[All Fields] OR "persistently"[All Fields] OR "persistents"[All Fields] OR "persister"[All Fields] OR "persisters"[All Fields] OR "persisting"[All Fields] OR "persists"[All Fields]) AND ("cell plasticity"[MeSH Terms] OR "cellular plasticity"[All Fields] OR "phenotypic plasticity"[All Fields] OR "plasticity"[All Fields])	None
PubMed	colorectal cancer EMT multidrug resistance	("colorectal neoplasms"[MeSH Terms] OR ("colorectal"[All Fields] AND "neoplasms"[All Fields]) OR "colorectal neoplasms"[All Fields] OR ("colorectal"[All Fields] AND "cancer"[All Fields]) OR "colorectal cancer"[All Fields]) AND ("epithelial-mesenchymal transition"[MeSH Terms] OR "epithelial mesenchymal transition"[All Fields] OR "EMT"[All Fields]) AND ("drug resistance, multiple"[MeSH Terms] OR ("drug"[All Fields] AND "resistance"[All Fields] AND "multiple"[All Fields]) OR "multiple drug resistance"[All Fields] OR ("multidrug"[All Fields] AND "resistance"[All Fields]) OR "multidrug resistance"[All Fields])	None
PubMed	colorectal cancer tumour microenvironment chemoresistance	("colorectal neoplasms"[MeSH Terms] OR ("colorectal"[All Fields] AND "neoplasms"[All Fields]) OR "colorectal neoplasms"[All Fields] OR ("colorectal"[All Fields] AND "cancer"[All Fields]) OR "colorectal cancer"[All Fields]) AND ("tumour microenvironment"[All Fields] OR "tumor microenvironment"[MeSH Terms] OR ("tumor"[All Fields] AND "microenvironment"[All Fields]) OR "tumor microenvironment"[All Fields]) AND ("chemoresistance"[All Fields] OR "chemoresistant"[All Fields] OR "chemoresistence"[All Fields] OR "chemoresistent"[All Fields])	None

Supplementary Table S1 and Supplementary Table S2

PubMed	colorectal cancer cancer associated fibroblasts chemoresistance	("colorectal neoplasms"[MeSH Terms] OR ("colorectal"[All Fields] AND "neoplasms"[All Fields]) OR "colorectal neoplasms"[All Fields] OR ("colorectal"[All Fields] AND "cancer"[All Fields]) OR "colorectal cancer"[All Fields]) AND ("cancer associated fibroblasts"[MeSH Terms] OR ("cancer associated"[All Fields] AND "fibroblasts"[All Fields]) OR "cancer associated fibroblasts"[All Fields] OR ("cancer"[All Fields] AND "associated"[All Fields] AND "fibroblasts"[All Fields]) OR "cancer associated fibroblasts"[All Fields]) AND ("chemoresistance"[All Fields] OR "chemoresistant"[All Fields] OR "chemoresistence"[All Fields] OR "chemoresistent"[All Fields])	None
PubMed	colorectal cancer immune suppression chemoresistance	("colorectal neoplasms"[MeSH Terms] OR ("colorectal"[All Fields] AND "neoplasms"[All Fields]) OR "colorectal neoplasms"[All Fields] OR ("colorectal"[All Fields] AND "cancer"[All Fields]) OR "colorectal cancer"[All Fields]) AND ("immunosuppression therapy"[MeSH Terms] OR ("immunosuppression"[All Fields] AND "therapy"[All Fields]) OR "immunosuppression therapy"[All Fields] OR ("immune"[All Fields] AND "suppression"[All Fields]) OR "immune suppression"[All Fields]) AND ("chemoresistance"[All Fields] OR "chemoresistant"[All Fields] OR "chemoresistence"[All Fields] OR "chemoresistent"[All Fields])	None
PubMed	colorectal cancer overcoming chemoresistance therapeutic strategies	("colorectal neoplasms"[MeSH Terms] OR ("colorectal"[All Fields] AND "neoplasms"[All Fields]) OR "colorectal neoplasms"[All Fields] OR ("colorectal"[All Fields] AND "cancer"[All Fields]) OR "colorectal cancer"[All Fields]) AND ("overcome"[All Fields] OR "overcomes"[All Fields] OR "overcoming"[All Fields]) AND ("chemoresistance"[All Fields] OR "chemoresistant"[All Fields] OR "chemoresistence"[All Fields] OR "chemoresistent"[All Fields]) AND ("drug discov today ther strateg"[Journal] OR ("therapeutic"[All Fields] AND "strategies"[All Fields]) OR "therapeutic strategies"[All Fields])	None
PMC	colorectal cancer 5-FU oxaliplatin irinotecan resistance	("colorectal neoplasms"[MeSH Terms] OR ("colorectal"[All Fields] AND "neoplasms"[All Fields]) OR "colorectal neoplasms"[All Fields] OR ("colorectal"[All Fields] AND "cancer"[All Fields]) OR "colorectal cancer"[All Fields]) AND ("fluorouracil"[Supplementary Concept] OR "fluorouracil"[All Fields] OR "5 fu"[All Fields] OR "fluorouracil"[MeSH Terms]) AND ("oxaliplatin"[Supplementary Concept] OR "oxaliplatin"[All Fields] OR "oxaliplatin"[MeSH Terms] OR "oxaliplatine"[All Fields] OR "oxaliplatin s"[All Fields]) AND ("irinotecan"[Supplementary Concept] OR "irinotecan"[All Fields] OR "irinotecan"[MeSH Terms] OR "irinotecan s"[All Fields]) AND ("resist"[All Fields] OR "resistance"[All Fields] OR "resistances"[All Fields] OR "resistant"[All Fields] OR "resistants"[All Fields] OR "resisted"[All Fields] OR "resistence"[All Fields] OR "resistences"[All Fields] OR "resistent"[All Fields] OR	None

Supplementary Table S1 and Supplementary Table S2

		"resistibility"[All Fields] OR "resisting"[All Fields] OR "resistive"[All Fields] OR "resistively"[All Fields] OR "resistivities"[All Fields] OR "resistivity"[All Fields] OR "resists"[All Fields])	
PMC	colorectal cancer Wnt beta-catenin chemoresistance	("colorectal neoplasms"[MeSH Terms] OR ("colorectal"[All Fields] AND "neoplasms"[All Fields]) OR "colorectal neoplasms"[All Fields] OR ("colorectal"[All Fields] AND "cancer"[All Fields]) OR "colorectal cancer"[All Fields]) AND "Wnt"[All Fields] AND ("ctnnb1 protein human"[Supplementary Concept] OR "ctnnb1 protein human"[All Fields] OR "beta catenin"[All Fields] OR "beta catenin"[Supplementary Concept] OR "beta catenin"[MeSH Terms] OR ("beta"[All Fields] AND "catenin"[All Fields])) AND ("chemoresistance"[All Fields] OR "chemoresistant"[All Fields] OR "chemoresistence"[All Fields] OR "chemoresistent"[All Fields])	None
PMC	colorectal cancer Notch YAP chemoresistance	("colorectal neoplasms"[MeSH Terms] OR ("colorectal"[All Fields] AND "neoplasms"[All Fields]) OR "colorectal neoplasms"[All Fields] OR ("colorectal"[All Fields] AND "cancer"[All Fields]) OR "colorectal cancer"[All Fields]) AND ("notch"[All Fields] OR "notch s"[All Fields] OR "notched"[All Fields] OR "notches"[All Fields] OR "notching"[All Fields] OR "notchings"[All Fields] OR "notchs"[All Fields]) AND "YAP"[All Fields] AND ("chemoresistance"[All Fields] OR "chemoresistant"[All Fields] OR "chemoresistence"[All Fields] OR "chemoresistent"[All Fields])	None
PMC	colorectal cancer tumour microenvironment chemoresistance	("colorectal neoplasms"[MeSH Terms] OR ("colorectal"[All Fields] AND "neoplasms"[All Fields]) OR "colorectal neoplasms"[All Fields] OR ("colorectal"[All Fields] AND "cancer"[All Fields]) OR "colorectal cancer"[All Fields]) AND ("tumour microenvironment"[All Fields] OR "tumor microenvironment"[MeSH Terms] OR ("tumor"[All Fields] AND "microenvironment"[All Fields]) OR "tumor microenvironment"[All Fields]) AND ("chemoresistance"[All Fields] OR "chemoresistant"[All Fields] OR "chemoresistence"[All Fields] OR "chemoresistent"[All Fields])	None

Supplementary Table S2. Structured evidence appraisal of all publications included in the review

Purpose and interpretation. This table applies the structured evidence framework described in Section 2.4 to the evidence used for mechanistic or therapeutic conclusions. Publications used only for epidemiological, clinical-treatment, pathway, methodological or other background context are retained for completeness but are marked “Not independently graded”. Evidence grades refer to the specific claim for which a publication is used in this review, not to the overall methodological quality of the publication.

Grading criteria. Strong: direct human/clinical validation of the specific resistance claim together with concordant mechanistic experimental evidence. **Moderate:** direct mechanistic evidence with functional validation across complementary CRC models, including organoid and/or in vivo

Supplementary Table S1 and Supplementary Table S2

evidence. **Limited:** direct evidence confined predominantly to cell-line/single-model studies, clinical association without functional causality, or incomplete functional validation. **Indirect:** evidence extrapolated from related biological, cancer or treatment contexts without direct demonstration of the specific acquired CRC chemotherapy-resistance mechanism. “Not established” is used in the main-text pathway table when no direct pathway–resistance relationship was identified; contextual sources in this supplementary table are instead marked “Not independently graded”.

Ref. / study	Study/source type	Treatment or resistance context	Role in this review	Specific claim supported	Direct relevance	Functional validation	Complementary / human validation	Evidence grade	Basis for grade / key limitation
[1] Bray et al., 2024	Epidemiological source	CRC epidemiology	Contextual evidence only	Global cancer incidence and mortality context	Context only	Not applicable for grading in this review	Not independently appraised for grade	Not independently graded	Used for clinical, biological, methodological or background context; did not independently increase a mechanistic or therapeutic evidence grade.
[2] Roshandel et al., 2024	Review/background	CRC epidemiology	Contextual evidence only	Epidemiology, risk factors and prevention	Context only	Not applicable for grading in this review	Not independently appraised for grade	Not independently graded	Used for clinical, biological, methodological or background context; did not independently increase a mechanistic or therapeutic evidence grade.
[3] Siegel et al., 2024	Epidemiological source	Cancer epidemiology	Contextual evidence only	Contemporary cancer statistics	Context only	Not applicable for grading in	Not independently apprais	Not independently	Used for clinical, biological, methodol

Supplementary Table S1 and Supplementary Table S2

						this review	ed for grade	graded	ogical or background context; did not independently increase a mechanistic or therapeutic evidence grade.
[4] de Gramont et al., 2000	Landmark clinical trial	FOLFOX/ CRC treatment	Contextual evidence only	Establishes fluorouracil/oxaliplatin clinical treatment context	Context only	Not applicable for grading in this review	Not independently appraised for grade	Not independently graded	Used for clinical, biological, methodological or background context; did not independently increase a mechanistic or therapeutic evidence grade.
[5] Tournigand et al., 2004	Landmark clinical trial	FOLFIRI/ FOLFOX	Contextual evidence only	Establishes standard treatment sequencing	Context only	Not applicable for grading in this review	Not independently appraised for grade	Not independently graded	Used for clinical, biological, methodological or background context; did not independently increase a mechanistic or therapeutic evidence grade.
[6] Cassidy et al., 2008	Clinical trial	CAPOX	Contextual evidence only	Establishes capecitabine/oxaliplatin	Context only	Not applicable for grading in	Not independently apprais	Not independently	Used for clinical, biological, methodol

Supplementary Table S1 and Supplementary Table S2

				treatment context		this review	ed for grade	grad ed	ogical or background context; did not independently increase a mechanistic or therapeutic evidence grade.
[7] Morris et al., 2023	Clinical guideline	Metastatic CRC	Contextual evidence only	Current systemic-treatment context	Context only	Not applicable for grading in this review	Not independently appraised for grade	Not independently graded	Used for clinical, biological, methodological or background context; did not independently increase a mechanistic or therapeutic evidence grade.
[8] Loree et al., 2017	Review	Metastatic CRC	Contextual evidence only	Treatment-development context	Context only	Not applicable for grading in this review	Not independently appraised for grade	Not independently graded	Used for clinical, biological, methodological or background context; did not independently increase a mechanistic or therapeutic evidence grade.
[9] Osterman et al., 2020	Clinical/epidemiological study	CRC recurrence	Contextual evidence only	Recurrence-risk context	Context only	Not applicable for grading in	Not independently apprais	Not independently	Used for clinical, biological, methodol

Supplementary Table S1 and Supplementary Table S2

						this review	ed for grade	graded	ogical or background context; did not independently increase a mechanistic or therapeutic evidence grade.
[10] Carlsen et al., 2021	Preclinical cell-based study	1. Introduction	Contextual evidence only	Used for contextual/background support in 1. Introduction.	Context only	Not applicable for grading in this review	Not independently appraised for grade	Not independently graded	Used for clinical, biological, methodological or background context; did not independently increase a mechanistic or therapeutic evidence grade.
[11] Blondy et al., 2020	Review	5-FU resistance	Contextual evidence only	Background synthesis of fluoropyrimidine-resistance mechanisms	Context only	Not applicable for grading in this review	Not independently appraised for grade	Not independently graded	Used for clinical, biological, methodological or background context; did not independently increase a mechanistic or therapeutic evidence grade.
[12] Martinez-Balibrea et	Review	Oxaliplatin resistance	Contextual evidence only	Background synthesis of platinum-	Context only	Not applicable for grading in	Not independently appraised	Not independently	Used for clinical, biological, methodol

al., 2015				resistance mechanisms		this review	ed for grade	graded	ogical or background context; did not independently increase a mechanistic or therapeutic evidence grade.
[13] Pires et al., 2010	Preclinical cell-based study	1. Introduction	Contextual evidence only	Used for contextual/background support in 1. Introduction.	Context only	Not applicable for grading in this review	Not independently appraised for grade	Not independently graded	Used for clinical, biological, methodological or background context; did not independently increase a mechanistic or therapeutic evidence grade.
[14] Gilbert et al., 2012	Review	Irinotecan/TOP1	Contextual evidence only	TOP1 biomarkers and therapeutic context	Context only	Not applicable for grading in this review	Not independently appraised for grade	Not independently graded	Used for clinical, biological, methodological or background context; did not independently increase a mechanistic or therapeutic evidence grade.
[15] Wood et al., 2015	Preclinical in vitro/in vivo study	1. Introduction	Contextual evidence only	Used for contextual/background support	Context only	Not applicable for grading in	Not independently apprais	Not independently	Used for clinical, biological, methodol

Supplementary Table S1 and Supplementary Table S2

				in 1. Introduction.		this review	ed for grade	graded	ogical or background context; did not independently increase a mechanistic or therapeutic evidence grade.
[16] De Angelis et al., 2006	Acquired 5-FU-resistant colon cancer cell lines	5-FU resistance	Graded mechanistic/therapeutic evidence	Stable 5-FU-resistant cells show altered treatment and recovery responses.	Yes	Drug exposure, withdrawal and recovery/rechallenge-type assessment.	Cell-line models only.	Limited	Direct acquired-resistance evidence, but validation is confined to cell models.
[17] Molavand et al., 2025	Review/contextual synthesis	CRC chemoresistance	Contextual evidence only	Broad drug-resistance and signalling context.	Context only	Not applicable for grading in this review	Not independently appraised for grade	Not independently graded	Used for contextual/background support only.
[18] Qiu et al., 2026	Review/contextual synthesis	CRC chemoresistance	Contextual evidence only	Broad contemporary resistance-mechanism synthesis.	Context only	Not applicable for grading in this review	Not independently appraised for grade	Not independently graded	Used for contextual/background support only.
[19] Zhang et al., 2008	Review	5-FU mechanism/resistance	Contextual evidence only	Pharmacological and molecular background for 5-FU resistance.	Context only	Not applicable for grading in this review	Not independently appraised for grade	Not independently graded	Used for background synthesis only.
[20] Peters et al., 2002	Review	5-FU/TYMS	Contextual evidence only	TYMS and fluoropyrimidine-response context.	Context only	Not applicable for grading in this review	Not independently appraised for grade	Not independently graded	Used for clinical/mechanistic context rather than independent grading.

Supplementary Table S1 and Supplementary Table S2

[21] Verma et al., 2022	Review	5-FU/DPD	Contextual evidence only	DPD-mediated fluoropyrimidine metabolism/resistance context.	Context only	Not applicable for grading in this review	Not independently appraised for grade	Not independently graded	Secondary evidence only.
[22] Gmeiner et al., 2023	Review/contextual synthesis	5-FU metabolism	Contextual evidence only	Fluoropyrimidine pharmacology and resistance context.	Context only	Not applicable for grading in this review	Not independently appraised for grade	Not independently graded	Secondary contextual source only.
[23] Zhang et al., 2020	CRC cell, mouse and human translational study	5-FU resistance; DPD-mediated drug catabolism	Graded mechanistic/therapeutic evidence	SphK2 promotes H3K56ac-mediated DPD expression and 5-FU catabolism, thereby conferring 5-FU resistance; SphK2 inhibition reverses resistance in experimental CRC models.	Yes	SphK2 genetic/pharmacological manipulation, ChIP-seq/luciferase assays and 5-FU response testing.	CRC cell lines, transgenic/knockout and xenograft mouse models, plus human CRC tissue showing SphK2–DPD association.	Moderate	Direct functional resistance mechanism validated across complementary in vitro and in vivo CRC models; human tissue supports the molecular association but does not provide direct treatment-outcome validation.
[24] Wilson et al., 2008	Review/perspective	5-FU/nucleotide metabolism	Contextual evidence only	Nucleotide-metabolism and treatment-response context.	Context only	Not applicable for grading in this review	Not independently appraised for grade	Not independently graded	Background source only.
[25] Stravopodis et al., 2011	Non-CRC bladder cancer cell study	5-FU/TS-p53 apoptosis context	Graded supporting evidence	TS inhibition can trigger p53-dependent and independ	Partial/indirect	Functional perturbation in a non-CRC model.	Non-CRC cell model.	Indirect	Relevant to fluoropyrimidine/p53 biology, but not direct

Supplementary Table S1 and Supplementary Table S2

				ent apoptosis.					evidence of acquired CRC chemoresistance.
[26] Dominijanni et al., 2018	CRC cell study	5-FU/p53-dependent response	Graded mechanistic/therapeutic evidence	p53 status alters fluoropyrimidine response.	Yes	Treatment-response comparison across p53 contexts.	Cell models only.	Limited	Direct CRC drug-response evidence without complementary organoid, in vivo or clinical validation.
[27] Tae et al., 2026	5-FU-resistant CRC cells, patient-derived organoids and xenografts	Acquired 5-FU resistance	Graded mechanistic/therapeutic evidence	EHMT2-PPM1B signalling supports 5-FU resistance; EHMT2 inhibition restores sensitivity.	Yes	EHMT2 knockdown/pharmacological inhibition with sensitisation, cell-cycle and apoptosis assays.	Resistant CRC cells, PDOs, xenografts; patient-response association is supportive.	Moderate	Direct mechanism validated across complementary advanced models; therapeutic inhibition remains preclinical.
[28] Cai et al., 2025	5-FU-resistant CRC cell and in vivo models	5-FU resistance	Graded mechanistic/therapeutic evidence	UGDH promotes 5-FU resistance through ROS-PI3K/AKT-EEF1A2-PRDX1 signalling.	Yes	Stable knockdown/overexpression with ROS, apoptosis and 5-FU response assays.	In vitro and in vivo CRC models.	Moderate	Direct mechanistic perturbation with complementary in vivo validation.
[29] Seetharam et al., 2009	Review/contextual synthesis	Oxaliplatin resistance	Contextual evidence only	Oxaliplatin-resistance background.	Context only	Not applicable for grading in this review	Not independently appraised for grade	Not independently graded	Contextual secondary evidence only.
[30] Alcindor et al., 2011	Review/contextual synthesis	Oxaliplatin mechanism	Contextual evidence only	Platinum pharmacology and resistance	Context only	Not applicable for grading in	Not independently apprais	Not independently	Contextual secondary

Supplementary Table S1 and Supplementary Table S2

				background.		this review	ed for grade	graded	evidence only.
[31] Seetharam et al., 2010	Oxaliplatin-resistant CRC cell models	Oxaliplatin resistance	Graded mechanistic/therapeutic evidence	ERCC1 upregulation contributes to oxaliplatin resistance.	Yes	ERCC1 siRNA silencing restores oxaliplatin sensitivity.	Cell models only.	Limited	Direct functional evidence, but restricted to cellular models.
[32] Bohanes et al., 2011	Review/contextual synthesis	Oxaliplatin/DNA repair	Contextual evidence only	DNA-repair-associated platinum-resistance context.	Context only	Not applicable for grading in this review	Not independently appraised for grade	Not independently graded	Used for background synthesis only.
[33] Kim et al., 2009	Clinical CRC cohort	5-FU/oxaliplatin treatment response	Graded mechanistic/therapeutic evidence	ERCC1/TS/GSTπ expression is associated with treatment outcome.	Yes	Clinical association only.	Human clinical cohort.	Limited	Direct human treatment association, but no functional mechanistic validation.
[34] Gao et al., 2026	CRC cells, DTPs, organoids and in vivo tumour models	Oxaliplatin resistance	Graded mechanistic/therapeutic evidence	LIG1-dependent repair limits oxaliplatin response; LIG1 targeting with gambogenic acid restores sensitivity.	Yes	LIG1 targeting, DNA-repair assays and chemosensitivity testing.	Cells, DTPs, organoids and in vivo models.	Moderate	Direct functional evidence reproduced across several complementary preclinical systems.
[35] Lee et al., 2023	CRC cell models	Oxaliplatin response	Graded mechanistic/therapeutic evidence	Glutathione depletion sensitises CRC cells to oxaliplatin.	Yes	Pharmacological perturbation with chemosensitivity testing.	Cell models only.	Limited	Direct functional support without complementary advanced-model validation.
[36] Pedraz-Cuesta et	Drug-resistant CRC cell models	SN-38/oxaliplatin response	Graded mechanistic/therapeutic	Glutamate transport and glutathio	Yes	Transporter inhibition with drug-	Cell models only.	Limited	Direct cellular evidence but no complem

Supplementary Table S1 and Supplementary Table S2

al., 2015			evidence	ne availability influence oxaliplatin/SN-38-induced cell death.		response assays.			entary organoid, in vivo or clinical validation.
[37] Fu et al., 2024	Oxaliplatin-resistant CRC cells and in vivo models	Oxaliplatin resistance	Graded mechanistic/therapeutic evidence	AKR1C1-STAT3 signalling raises glutathione and drives oxaliplatin resistance.	Yes	Genetic silencing and pharmacological inhibition restore oxaliplatin sensitivity.	Cell models plus in vivo resistant-tumour models.	Moderate	Direct functional resistance mechanism with complementary in vivo validation.
[38] Buß et al., 2018	CRC cell models	Oxaliplatin uptake/resistance	Graded mechanistic/therapeutic evidence	CTR1 and organic cation transporters influence oxaliplatin uptake and resistance.	Yes	Transport/uptake and drug-response analyses.	Cell models only.	Limited	Direct transporter evidence, but confined to cellular models.
[39] Le Roy et al., 2016	Clinical case-control study	FOLFOX-4/oxaliplatin response	Graded mechanistic/therapeutic evidence	Tumour expression of oxaliplatin transporters is associated with response to adjuvant FOLFOX-4.	Yes	Clinical association; no experimental perturbation.	Human CRC treatment cohort.	Limited	Direct human treatment-response evidence, but mechanistic causality is not established.
[40] Martinez-Bernabe et al., 2024	CRC translational biomarker study	Oxaliplatin response/metabolic state	Graded mechanistic/therapeutic evidence	Oxidative-phosphorylation features are evaluated as predictors of oxaliplatin response.	Yes	Biomarker/response analysis.	Translational evidence; mechanistic perturbation is limited.	Limited	Relevant to treatment response, but functional validation of a resistance mechanism is incomplete.

Supplementary Table S1 and Supplementary Table S2

[41] Jeong et al., 2019	Oxaliplatin-resistant CRC cell models	Oxaliplatin resistance	Graded mechanistic/therapeutic evidence	Cannabidiol restores oxaliplatin response through NOS3/SOD2-associated autophagy.	Yes	Pharmacological treatment with mechanistic and chemosensitivity assays.	Predominantly cell-based evidence.	Limited	Direct resistance-reversal evidence, but complementary organoid/in vivo/clinical validation is limited in the evidence used here.
[42] Thomas et al., 2019	Review	Irinotecan resistance	Contextual evidence only	TOP1/irinotecan therapeutic background.	Context only	Not applicable for grading in this review	Not independently appraised for grade	Not independently graded	Contextual review only.
[43] Ozawa et al., 2021	Review/contextual synthesis	Irinotecan resistance	Contextual evidence only	Cellular irinotecan resistance and therapeutic-combination context.	Context only	Not applicable for grading in this review	Not independently appraised for grade	Not independently graded	Review article; did not independently contribute to an evidence grade.
[44] Gongora et al., 2011	Camptothecin-resistant cancer cell models	TOP1-mediated irinotecan-class resistance	Graded mechanistic/therapeutic evidence	TOP1 mutations are associated with resistance to camptothecin-class agents.	Partial	Resistant-model molecular characterisation.	Predominantly cell models; not a complementary CRC model series.	Limited	Direct target-based resistance evidence, but limited model breadth and CRC-specific translational validation.
[45] Jensen et al., 2016	SN-38-resistant human colon cancer cell lines	Irinotecan/SN-38 resistance	Graded mechanistic/therapeutic evidence	Resistance-associated TOP1 mutations occur in SN-38-resistant colon	Yes	Established acquired-resistant cell models with TOP1 characterisation.	Cell models only.	Limited	Direct acquired-resistance evidence without organoid, in vivo or clinical

Supplementary Table S1 and Supplementary Table S2

				cancer cells.					validation.
[46] Nielsen et al., 2017	Review	ABCG2/irinotecan	Contextual evidence only	Context for transporter-mediated irinotecan response.	Context only	Not applicable for grading in this review	Not independently appraised for grade	Not independently graded	Review article used for contextual synthesis only.
[47] de Man et al., 2018	Review/contextual synthesis	Irinotecan pharmacology	Contextual evidence only	Pharmacokinetic, pharmacodynamic and pharmacogenetic context for irinotecan.	Context only	Not applicable for grading in this review	Not independently appraised for grade	Not independently graded	Review article; did not independently increase a mechanistic evidence grade.
[48] Wang et al., 2012	Clinical CRC cohort	Irinotecan/5-FU pharmacogenetics	Graded mechanistic/therapeutic evidence	UGT1A1 genotype is associated with outcomes during irinotecan-based treatment.	Partial	Clinical association only.	Human treatment cohort.	Indirect	Clinically relevant to irinotecan exposure/outcome, but does not directly establish tumour-cell acquired resistance.
[49] Jossé et al., 2014	Cancer cell and in vivo models	TOP1-inhibitor response	Graded supporting evidence	ATR inhibition increases sensitivity to TOP1 inhibition/irinotecan.	Partial/indirect	Pharmacological ATR inhibition.	In vivo validation.	Indirect	Evidence is extrapolated from a related biological/treatment context rather than directly demonstrating the specific acquired CRC pathway-resistance mechanism.

Supplementary Table S1 and Supplementary Table S2

[50] Manic et al., 2021	Primary CRC stem-cell and in vivo models	Replication-stress survival	Graded mechanistic/therapeutic evidence	PARP1-MRE11-RAD51 programmes support replication-stress tolerance in CRC stem cells.	Partial	PARP1/MRE11/RAD51 perturbation with sensitisation assays.	Primary CRC stem-cell models with complementary functional testing.	Indirect	Strong CRC replication-stress biology, but no direct demonstration of the specific irinotecan/Wnt/Notch/YAP relationship graded in Table 2.
[51] Lin et al., 2025	FBW7-deficient CRC cells, patient tissue and xenografts	Irinotecan/SN-38 resistance	Graded mechanistic/therapeutic evidence	FBW7 deficiency stabilises MCL1 and drives irinotecan resistance; maritoclax restores sensitivity.	Yes	CRISPR/RNAi, MCL1 manipulation and pharmacological resensitisation.	CRC patient tissue plus in vivo xenografts.	Moderate	Direct mechanism with complementary in vivo and supportive human tissue evidence; the therapeutic intervention remains preclinical.
[52] Chen et al., 2022	Mini-review	CRC CSC chemoresistance	Contextual evidence only	CSC-related chemoresistance context	Context only	Not applicable for grading in this review	Not independently appraised for grade	Not independently graded	Secondary contextual source only.
[53] Begicevic et al., 2017	Review	ABC transporters/CSCs	Contextual evidence only	ABC-transporter contribution to CSC drug resistance.	Context only	Not applicable for grading in this review	Not independently appraised for grade	Not independently graded	Secondary contextual source only.
[54] Hsu et al., 2018	Oxaliplatin-resistant CRC cell models	Oxaliplatin/cross-resistance	Graded mechanistic/therapeutic evidence	ABCG2 activation reduces ER-stress-mediated apoptosis and contribute	Yes	ABCG2/pathway manipulation and chemosensitivity testing.	Cell models only.	Limited	Direct functional evidence, but no higher-order

				s to oxaliplatin resistance.					validation.
[55] Zhong et al., 2024	Review	CRC stem cells	Contextual evidence only	CSC contribution to drug resistance	Context only	Not applicable for grading in this review	Not independently appraised for grade	Not independently graded	Secondary contextual source only.
[56] Garza-Treviño et al., 2015	Review	Colon CSCs	Contextual evidence only	CSC biology and therapeutic context	Context only	Not applicable for grading in this review	Not independently appraised for grade	Not independently graded	Secondary contextual source only.
[57] Escalante et al., 2021	Review	EMT/FOLFOX resistance	Contextual evidence only	EMT-associated resistance context	Context only	Not applicable for grading in this review	Not independently appraised for grade	Not independently graded	Secondary contextual source only.
[58] Sun et al., 2017	CRC cell lines plus chemotherapy-treated stage II/III patient cohort	5-FU resistance; Notch/HES1	Graded mechanistic/therapeutic evidence	HES1 promotes 5-FU resistance through EMT and ABC-transporter induction.	Yes	HES1 overexpression/inhibition with cytotoxicity and molecular assays.	Human adjuvant-chemotherapy cohort linked HES1 to recurrence.	Limited	Direct functional evidence in CRC cell models with supportive retrospective clinical association, but the human data do not directly validate HES1-mediated 5-FU resistance causally.
[59] Jiang et al., 2017	Review	EMT/ABC transporters	Contextual evidence only	EMT–transporter relationship	Context only	Not applicable for grading in this review	Not independently appraised for grade	Not independently graded	Secondary contextual source only.
[60] Fodde, 2002	Review	APC	Contextual evidence only	APC biology in CRC	Context only	Not applicable for grading in	Not independently apprais	Not independently	Foundational backgrou

Supplementary Table S1 and Supplementary Table S2

						this review	ed for grade	graded	nd source only.
[61] Schatoff et al., 2017	Review	Wnt/CRC	Contextual evidence only	Canonical Wnt pathway background	Context only	Not applicable for grading in this review	Not independently appraised for grade	Not independently graded	Foundational background source only.
[62] Hankey et al., 2018	Review	APC/Wnt	Contextual evidence only	APC-dependent and independent signalling	Context only	Not applicable for grading in this review	Not independently appraised for grade	Not independently graded	Background source only.
[63] He et al., 1998	Foundational mechanistic study	c-MYC/Wnt	Contextual evidence only	Establishes c-MYC as an APC/β-catenin target	Context only	Not applicable for resistance grading	Not independently appraised for grade	Not independently graded	Foundational pathway evidence, not acquired chemoresistance.
[64] Shtutman et al., 1999	Foundational mechanistic study	Cyclin D1/Wnt	Contextual evidence only	Establishes cyclin D1 as a β-catenin target	Context only	Not applicable for resistance grading	Not independently appraised for grade	Not independently graded	Foundational pathway evidence, not acquired chemoresistance.
[65] Brabletz et al., 2000	Human pathology study	β-catenin/c-MYC	Contextual evidence only	Human CRC Wnt-expression context	Context only	Not applicable for resistance grading	Human tumour specimens	Not independently graded	Provides pathway context rather than evidence for a specific acquired resistance claim.
[66] Chikazawa et al., 2010	Chemotherapy-resistant side-population colon cancer cells	Wnt-associated multidrug resistance	Graded mechanistic/therapeutic evidence	Wnt inhibition reduces chemotherapy-resistant side-population cells.	Yes	Wnt-pathway inhibition with resistant-population assessment.	Cell models only.	Limited	Direct pathway-related evidence, but limited to cellular models.
[67] Liu et al., 2021	CRC cells, in vivo models and chemoresistant	Wnt/stemness-associated chemoresistance	Graded mechanistic/therapeutic evidence	Sec62 promotes CRC stemness and chemoresistance	Yes	Sec62 depletion/overexpression and pathway assays.	In vivo models and human chemoresistant	Strong	Functional mechanism supported by complem

Supplementary Table S1 and Supplementary Table S2

	human CRC tissues			through Wnt/β-catenin signalling.			CRC tissue.		entary in vivo and direct human chemoresistance evidence.
[68] Chen et al., 2019	CRC experimental study	Wnt/stemness context	Graded supporting evidence	miR-103/107 prolongs Wnt/β-catenin signalling and CRC stemness via AXIN2.	Partial/indirect	Genetic manipulation.	Experimental CRC models; no direct acquired drug-resistance demonstration.	Indirect	Supports Wnt/stemness biology, but not a specific acquired chemotherapy-resistance mechanism.
[69] Stein et al., 2012	Colon cancer cell and human tumour analysis	β-catenin/ABCB1 drug-efflux biology	Graded mechanistic/therapeutic evidence	Mutant β-catenin regulates ABCB1 expression.	Partial	β-catenin manipulation and promoter studies.	Human tumour expression analysis; drug-specific resistance effect was not firmly established.	Indirect	Mechanistic ABCB1 regulation is shown, but the specific irinotecan-resistance relationship remains indirect.
[70] Wang et al., 2015	5-FU-resistant CRC cell models	5-FU resistance	Graded mechanistic/therapeutic evidence	TRPC5/Wnt-associated signalling contributes to 5-FU resistance and its inhibition restores sensitivity.	Yes	TRPC5 inhibition with 5-FU resensitisation assays.	Predominantly cell models.	Limited	Direct functional drug-resistance evidence without complementary advanced-model validation.
[71] Lapham et al., 2009	CRC cell study	Wnt/apoptosis context	Graded supporting evidence	β-catenin/TCF4 transcriptionally activates BCL-W.	Partial/indirect	Promoter/pathway manipulation.	Cell models only.	Indirect	Establishes Wnt-BCL-W biology, but not direct acquired 5-FU resistance.

Supplementary Table S1 and Supplementary Table S2

[72] Pate et al., 2014	CRC metabolic/pathway study	Wnt/metabolic adaptation context	Graded supporting evidence	Wnt signalling directs glycolytic/angiogenic metabolic programmes.	Partial/indirect	Wnt/metabolic manipulation.	Experimental CRC models.	Indirect	Supports metabolic consequences of Wnt, not a drug-specific acquired-resistance phenotype.
[73] Wang et al., 2017	CRC cell and clinical tissue association study	5-FU chemoresistance/metabolic adaptation	Graded mechanistic/therapeutic evidence	TRPC5 and GLUT1 expression is associated with CRC chemoresistance.	Yes	Expression and treatment-response association; mechanistic perturbation is limited in this paper.	Human CRC tissue association.	Limited	Human association supports relevance, but direct functional causality is incomplete.
[74] Yuan et al., 2020	Review	Wnt/CRC resistance	Contextual evidence only	Wnt-associated stemness and treatment-survival context.	Context only	Not applicable for grading in this review	Not independently appraised for grade	Not independently graded	Used for contextual/background support only.
[75] Ayadi et al., 2015	Multiple acquired chemotherapy-resistant CRC cell lines	Chronic chemotherapy/Wnt-stemness adaptation	Graded mechanistic/therapeutic evidence	Chronic chemotherapeutic stress can alter stemness and Wnt/β-catenin signalling in resistant CRC cells.	Yes	Long-term acquired-resistance models with pathway inhibition.	Cell models only.	Limited	Direct acquired-resistance modelling, but pathway inhibition did not establish a universal Wnt dependency and no advanced-model validation was provided.
[76] Touil et al., 2014	Acquired 5-FU-resistant CRC cells plus human post-5-FU liver	5-FU quiescent/DTP-like survival; c-Yes/YAP	Graded mechanistic/therapeutic evidence	c-Yes/YAP-associated reversible quiescence supports survival	Yes	YES1/YAP functional manipulation and treatment/re-exposure analysis.	Human liver metastases after 5-FU-based neoadjuvant	Limited	Direct functional evidence for a reversible treatment-surviving

Supplementary Table S1 and Supplementary Table S2

	metastases			after 5-FU and relates to relapse.			chemotherapy.		state with supportive treatment-exposed human material, but the human evidence does not directly establish c-Yes/YAP1-mediated reversible quiescence as a stable acquired-resistance mechanism.
[77] Park et al., 2021	CRC cells, mouse models and patient tumours after chemoradiation	Wnt-associated therapy resistance	Graded mechanistic/therapeutic evidence	CD45-Wnt signalling promotes stemness and survival after 5-FU/chemoradiation.	Yes	CD45 depletion and pathway assays with therapy-response testing.	Mouse models and human pretreated tumour specimens.	Strong	Functional mechanism with direct validation in treatment-exposed human CRC material.
[78] He et al., 2018	5-FU-resistant CRC cell and xenograft models	5-FU/Wnt resistance	Graded mechanistic/therapeutic evidence	Wnt/β-catenin-CHK1 signalling contributes to 5-FU resistance.	Yes	Wnt and CHK1 manipulation with chemosensitivity testing.	In vivo xenograft validation.	Moderate	Direct functional evidence with complementary in vivo validation; no human or clinical validation.
[79] Liu et al., 2021	CRC cells and xenografts	Oxaliplatin resistance	Graded mechanistic/therapeutic evidence	SOX9-MMS22L-dependent DNA repair promotes oxaliplati	Yes	SOX9/MMS22L overexpression/knockdown, DNA-repair and	In vivo xenograft validation; human CRC	Moderate	Direct functional mechanism with complementary in

Supplementary Table S1 and Supplementary Table S2

				n resistance.		chemosensitivity assays.	expression/prognostic association.		vivo validation; human data are supportive rather than direct treatment-validation.
[80] Huang et al., 2024	Oxaliplatin-resistant CRC models with in vivo validation	Oxaliplatin resistance	Graded mechanistic/therapeutic evidence	Wnt/β-catenin-IMPDH2-driven purine metabolism promotes oxaliplatin resistance.	Yes	Wnt/IMPDH2 manipulation, metabolic rescue and chemosensitivity assays.	Complementary in vivo CRC models.	Moderate	Direct functional mechanism reproduced beyond cell culture.
[81] Betson et al., 2022	Multiple CRC cell lines	Chemotherapy response/ABC transporters	Graded mechanistic/therapeutic evidence	IGF2BP1 supports ABCB1/ABCG2 expression and chemoresistance, including irinotecan response.	Yes	IGF2BP1 knockdown with chemosensitivity/apoptosis assays.	Cell models only.	Limited	Direct functional evidence but no organoid, in vivo or human validation.
[82] Shah et al., 2021	Review	PORCN inhibitors	Contextual evidence only	Wnt-targeted therapeutic context	Context only	Not applicable for grading in this review	Not independently appraised for grade	Not independently graded	Secondary contextual source only.
[83] Rodon et al., 2021	Phase I study	WNT974	Contextual evidence only	Clinical Wnt-inhibitor safety/activity context	Context only	Not applicable for resistance grading	Human phase I trial	Not independently graded	Used to establish pathway-targeting feasibility rather than acquired chemotherapy-resistance evidence.
[84] Flanagan et al., 2022	Review	PORCN inhibition	Contextual evidence only	Ligand-dependent Wnt targeting	Context only	Not applicable for grading in	Not independently apprais	Not independently	Secondary contextual source only.

Supplementary Table S1 and Supplementary Table S2

						this review	ed for grade	grad ed	
[85] Madan et al., 2016	Preclinical cancer study	Wnt-targeted therapy context	Graded supporting evidence	PORCN inhibition suppresses ligand-dependent Wnt-driven tumours.	Partial/indirect	Pharmacological Wnt inhibition.	In vivo models.	Indirect	Pathway-targeting evidence without direct acquired CRC chemotherapy-resistance modelling.
[86] Bond et al., 2016	Human CRC molecular study	Wnt-targeting stratification	Graded supporting evidence	RNF43/ZNRF3 alterations define ligand-dependent Wnt contexts.	Partial/indirect	Molecular association.	Human tumours.	Indirect	Relevant to pathway stratification, not direct acquired chemoresistance.
[87] Huels et al., 2018	Intestinal/CRC in vivo study	Wnt biology/targeting	Graded supporting evidence	Wnt ligands influence tumour initiation and stem-cell competition.	Partial/indirect	Genetic/pathway manipulation.	In vivo models.	Indirect	Supports pathway biology and targeting limitations rather than acquired chemotherapy resistance.
[88] Tabernero et al., 2023	Phase Ib/II study	WNT974 combinations	Contextual evidence only	Clinical Wnt-targeting context	Context only	Not applicable for resistance grading	Human clinical trial	Not independently graded	Used for clinical pathway-targeting context, not direct acquired chemoresistance evidence.
[89] Nachef et al., 2026	Review	Wnt inhibitors	Contextual evidence only	Bone/on-target toxicity	Context only	Not applicable for grading in this review	Not independently appraised for grade	Not independently graded	Secondary contextual source only.
[90] Shin et al., 2017	CRC in vitro/in vivo study	Wnt-targeted therapy	Graded supporting evidence	β-catenin-TCF4 inhibition suppresse	Partial/indirect	Direct pathway inhibition.	Cell and in vivo models.	Indirect	Demonstrates Wnt druggability but not

Supplementary Table S1 and Supplementary Table S2

				s CRC growth.					acquired chemotherapy resistance.
[91] Tanton et al., 2022	CRC preclinical study	Wnt-targeted therapy	Graded supporting evidence	β-catenin/BCL9 inhibition suppresses oncogenic Wnt signalling.	Partial/indirect	Pharmacological pathway inhibition.	Preclinical CRC models.	Indirect	Therapeutic pathway evidence without direct acquired chemotherapy-resistance modelling.
[92] Chen et al., 2023	Review	Wnt targeting	Contextual evidence only	Strategies for Wnt inhibition in CRC	Context only	Not applicable for grading in this review	Not independently appraised for grade	Not independently graded	Secondary contextual source only.
[93] Kahn, 2014	Review	Wnt safety	Contextual evidence only	Safety limitations of Wnt targeting	Context only	Not applicable for grading in this review	Not independently appraised for grade	Not independently graded	Secondary contextual source only.
[94] Ji et al., 2022	Translational modelling	WNT974	Contextual evidence only	Dose-selection/translational context	Context only	Not applicable for resistance grading	Translational pharmacological modelling	Not independently graded	Used for Wnt-inhibitor development context rather than a resistance claim.
[95] Cho et al., 2020	Mouse CRC models, patient-derived tumour organoids and patient-derived tumour cells	5-FU-induced stemness/tumour repopulation	Graded mechanistic/therapeutic evidence	5-FU activates p53-WNT3-β-catenin signalling, enriching residual stem-like cells and promoting regrowth.	Yes	Pathway manipulation and Wnt inhibition with treatment/withdrawal response.	Mouse models plus patient-derived organoid/cell models.	Moderate	Direct functional chemotherapy-adaptation evidence across complementary advanced models; no prospective clinical validation.

Supplementary Table S1 and Supplementary Table S2

[96] Wang et al., 2018	Acquired 5-FU-resistant CRC cells plus human chemotherapy-outcome cohort	5-FU resistance; TRPC5/GLUT1-dependent glycolytic adaptation	Graded mechanistic/therapeutic evidence	Enhanced glycolysis supports TRPC5-associated 5-FU resistance; glycolysis inhibition reverses resistance, and high TRPC5/GLUT1 expression is associated with poorer chemotherapy outcome.	Yes	TRPC5 knockdown and pharmacological glycolysis inhibition with ATP, apoptosis and 5-FU sensitivity assays.	5-FU-resistant CRC cell models plus human CRC tissue/clinical outcome association.	Strong	Direct functional 5-FU-resistance evidence with concordant human chemotherapy-outcome association, although the clinical evidence is observational rather than prospective.
[97] Tyagi et al., 2020	Review/contextual synthesis	Notch/CRC	Contextual evidence only	Notch-associated stemness, survival and therapeutic context.	Context only	Not applicable for grading in this review	Not independently appraised for grade	Not independently graded	Used for contextual/background support only.
[98] van Es et al., 2005	Foundational study	Notch	Contextual evidence only	Intestinal differentiation and Notch biology	Context only	Not applicable for grading in this review	Not independently appraised for grade	Not independently graded	Foundational pathway context.
[99] Sikandar et al., 2010	Experimental/contextual source	Notch/CSC	Contextual evidence only	Tumour-initiating-cell and differentiation biology	Context only	Not applicable for resistance grading	Experimental CRC models	Not independently graded	Used to establish Notch/CSC biology rather than direct chemotherapy resistance.
[100] Lu et al., 2013	CRC endothelial/stem-cell experimental study	Notch/TME stemness	Graded supporting evidence	Endothelial Jagged1 promotes a CRC stem-cell phenotype.	Partial/indirect	Jagged1/Notch functional manipulation.	Experimental/in vivo CRC models.	Indirect	Supports TME-Notch stemness biology, not a direct acquired chemothe

Supplementary Table S1 and Supplementary Table S2

									rapy-resistance mechanism.
[101] Radu et al., 2024	Review	CRC CSCs	Contextual evidence only	Cancer-stem-cell context	Context only	Not applicable for grading in this review	Not independently appraised for grade	Not independently graded	Secondary source only.
[102] Fender et al., 2015	CRC cell study	Notch/EMT/stemness	Graded supporting evidence	Notch1 promotes stemness and EMT in CRC.	Partial/indirect	Notch manipulation.	Cell models.	Indirect	Adaptive-state biology without drug-specific acquired resistance.
[103] Pal et al., 2018	Metastatic colon cancer preclinical study	Notch/AKT/EMT	Graded supporting evidence	Notch1/AKT inhibition suppresses EMT.	Partial/indirect	Functional pathway inhibition.	Preclinical models.	Indirect	Related pathway evidence without direct chemotherapy-resistance demonstration.
[104] Vermeulen et al., 2010	CRC stem-cell translational study	Wnt/stemness	Graded supporting evidence	Wnt activity defines colon CSCs and is microenvironment-regulated.	Partial/indirect	Functional stem-cell assays.	Human/in vivo support.	Indirect	Foundational stemness evidence, not direct acquired chemotherapy resistance.
[105] Rodilla et al., 2009	CRC experimental study	Wnt-Notch crosstalk	Graded supporting evidence	Jagged1 links Wnt and Notch pathways in CRC.	Partial/indirect	Genetic/pathway manipulation.	Preclinical CRC models.	Indirect	Establishes pathway crosstalk but not a drug-specific resistance phenotype.
[106] Pannequin et al., 2009	CRC cell study	Wnt-Notch crosstalk	Graded supporting evidence	Wnt target Jagged1 mediates Notch activation by	Partial/indirect	Pathway manipulation.	Cell models.	Indirect	Mechanistic crosstalk evidence without direct acquired

Supplementary Table S1 and Supplementary Table S2

				progastrin.					chemoresistance.
[107] Bertrand et al., 2012	Review	Developmental pathways	Contextual evidence only	Wnt/Notch/BMP/Hedgehog crosstalk	Context only	Not applicable for grading in this review	Not independently appraised for grade	Not independently graded	Secondary contextual source only.
[108] Meng et al., 2009	Colon cancer cell models	Oxaliplatin/Notch adaptation	Graded mechanistic/therapeutic evidence	Oxaliplatin activates Notch1; γ-secretase inhibition enhances chemosensitivity.	Yes	γ-secretase inhibition and NICD manipulation with drug-response assays.	Cell models only.	Limited	Direct drug-specific functional evidence without organoid, in vivo or clinical validation.
[109] Wang et al., 2020	CRC cells, human CRC specimens and patient-derived xenografts	Oxaliplatin resistance	Graded mechanistic/therapeutic evidence	Low H3K27me3/NOTCH2-associated adaptation contributes to oxaliplatin resistance and can be therapeutically reversed.	Yes	Epigenetic manipulation with apoptosis/chemosensitivity assays.	Human chemotherapy-outcome association and oxaliplatin-resistant PDX validation.	Moderate	Direct functional resistance evidence with complementary PDX validation; the human chemotherapy-outcome association is supportive but does not constitute direct clinical validation of the proposed mechanism.
[110] Mouillet-Richard et al., 2020	Review	YAP/TAZ	Contextual evidence only	YAP/TAZ and CRC molecular subtypes	Context only	Not applicable for grading in this review	Not independently appraised for grade	Not independently graded	Secondary contextual source only.
[111] Zinatizadeh	Review	Hippo/YAP	Contextual	Hippo pathway and	Context only	Not applicable for	Not independently	Not independ	Secondary contextua

Supplementary Table S1 and Supplementary Table S2

et al., 2019			evidence only	cancer progression		grading in this review	appraised for grade	ently graded	l source only.
[112] Mohammadpour et al., 2024	Review	Hippo/CRC	Contextual evidence only	Hippo signalling and therapeutic potential	Context only	Not applicable for grading in this review	Not independently appraised for grade	Not independently graded	Secondary contextual source only.
[113] Huang et al., 2020	Acquired 5-FU-resistant CRC cells and xenograft models	5-FU resistance	Graded mechanistic/therapeutic evidence	Combined EGFR and YAP inhibition reverses 5-FU resistance.	Yes	YAP/EGFR inhibition with chemosensitivity assays.	Cell models plus in vivo xenografts; no direct clinical validation.	Moderate	Direct functional resistance mechanism reproduced in vitro and in vivo.
[114] Yang et al., 2025	Oxaliplatin-resistant CRC cells and xenografts	Oxaliplatin/YAP resistance	Graded mechanistic/therapeutic evidence	EDN1-β-arrestin1-YAP signalling drives oxaliplatin resistance.	Yes	EDN1 knockdown/overexpression and YAP-pathway manipulation with chemosensitivity assays.	In vivo xenograft validation; human survival association is supportive.	Moderate	Direct functional mechanism with complementary in vivo validation; human data do not directly validate treatment resistance.
[115] Xu et al., 2024	CRC cells, xenografts and human CRC tissue	5-FU/YAP metabolic resistance	Graded mechanistic/therapeutic evidence	YAP promotes 5-FU chemoresistance through the mTOR-GLUT3 axis.	Yes	YAP/mTOR/GLUT3 manipulation with chemosensitivity and metabolic assays.	In vivo xenografts; human tissue/prognostic association.	Moderate	Direct functional mechanism with in vivo validation; human evidence is supportive rather than treatment-specific.
[116] Kim et al., 2026	5-FU- and oxaliplatin-resistant	YAP-linked chemoresistance	Graded mechanistic/therapeutic evidence	AMOT-p80 supports YAP-dependent	Yes	AMOT depletion, isoform re-expression/rescue and YAP	Cell models only.	Limited	Strong cell-level causality, but no organoid, in vivo or

Supplementary Table S1 and Supplementary Table S2

	CRC cell models			resistance to 5-FU and oxaliplatin.		manipulation.			human validation.
[117] Nguyen et al., 2019	Review	Notch/Hippo-YAP and resistance	Contextual evidence only	General signalling and resistance context.	Context only	Not applicable for grading in this review	Not independently appraised for grade	Not independently graded	Used for contextual/background support only.
[118] Zeng et al., 2021	Review	Hippo/drug resistance	Contextual evidence only	YAP/TAZ drug-resistance context	Context only	Not applicable for grading in this review	Not independently appraised for grade	Not independently graded	Secondary contextual source only.
[119] Ogasawara et al., 2024	CRC tumour-line study	YAP/plasticity	Graded supporting evidence	Non-genomic drivers modulate YAP signalling and CRC plasticity.	Partial/indirect	Molecular perturbation/characterisation.	CRC tumour-line models.	Indirect	Supports plasticity/YAP biology, but not a specific cytotoxic drug-resistance mechanism.
[120] Brosseau et al., 2025	Lung cancer experimental study	YAP/chemoresistance cross-cancer context	Graded supporting evidence	YAP/TEAD contributes to paclitaxel resistance.	Partial/indirect	Functional pathway studies.	Non-CRC model.	Indirect	Cross-cancer chemotherapy evidence only.
[121] Edwards et al., 2023	Cancer/CRC targeted-therapy study	KRASG12C inhibitor resistance	Graded supporting evidence	YAP1/TAZ drives primary/acquired resistance to KRASG12C inhibition.	Partial/indirect	TEAD/YAP pathway inhibition and resistance assays.	Targeted-therapy context.	Indirect	Resistance biology is relevant to adaptive signalling, but treatment context is not cytotoxic CRC chemotherapy.
[122] Xiang et al., 2025	CRC organoids and patient-derived orthotopic	FOLFOX adaptive resistance	Graded mechanistic/therapeutic evidence	ADAM10-NOTCH2-TCF7L2-mediated Wnt transcription	Yes	Genetic/pharmacological pathway disruption and chemotherapy-	Organoid and PDOX validation.	Moderate	Direct functional mechanism reproduced across complem

Supplementary Table S1 and Supplementary Table S2

	xenograft models			supports FOLFOX resistance; pathway disruption restores sensitivity.		response testing.			entary advanced CRC models.
[123] Bhavanasi et al., 2016	Review	Wnt/stem cells	Contextual evidence only	Wnt signalling in normal/malignant stem cells	Context only	Not applicable for grading in this review	Not independently appraised for grade	Not independently graded	Secondary contextual source only.
[124] Herbst et al., 2014	CRC cell transcriptomic study	β-catenin target-gene context	Graded supporting evidence	Defines β-catenin target genes in CRC cells.	Partial/indirect	Transcriptomic/pathway characterisation.	Cell models.	Indirect	Foundational pathway evidence rather than acquired chemoresistance.
[125] Konsavage et al., 2012	CRC cell and human tumour pathway study	Wnt/β-catenin–YAP1 crosstalk	Graded supporting evidence	β-catenin/TCF4 directly drives YAP1 gene expression in CRC cells, providing a mechanistic interface between Wnt/β-catenin and Hippo/YAP1 signalling.	Partial/indirect	β-catenin knockdown and enhancer/transcriptional analyses of YAP1 regulation.	CRC cell models plus primary human colorectal tumour expression/localisation data.	Indirect	Establishes direct pathway crosstalk in CRC but does not demonstrate a drug-specific acquired-chemoresistance phenotype.
[126] Cammarer i et al., 2025	CRC experimental/translational study	Multilineage plasticity	Graded supporting evidence	Loss of colonic fidelity enables multilineage plasticity and metastatic progression.	Partial/indirect	Functional lineage-state studies.	Advanced CRC models/human relevance.	Indirect	Supports plasticity biology rather than a specific chemotherapy-resistance mechanism.

Supplementary Table S1 and Supplementary Table S2

[127] Angius et al., 2021	Review	CRC CSCs	Contextual evidence only	Molecular markers and pathways	Context only	Not applicable for grading in this review	Not independently appraised for grade	Not independently graded	Secondary contextual source only.
[128] Sphyris et al., 2021	Review	Stem-cell niches	Contextual evidence only	Niche signalling in CRC	Context only	Not applicable for grading in this review	Not independently appraised for grade	Not independently graded	Secondary contextual source only.
[129] Sprangers et al., 2021	Review	Organoids	Contextual evidence only	Organoid modelling of intestinal biology	Context only	Not applicable for grading in this review	Not independently appraised for grade	Not independently graded	Secondary contextual source only.
[130] Swayden et al., 2020	Review	1. Introduction; 5.4. Therapy-Induced Signalling Adaptation, Cellular Plasticity and Drug-Tolerant Persister States	Contextual evidence only	Used for contextual/background support in 1. Introduction; 5.4. Therapy-Induced Signalling Adaptation, Cellular Plasticity and Drug-Tolerant Persister States.	Context only	Not applicable for grading in this review	Not independently appraised for grade	Not independently graded	Used for clinical, biological, methodological or background context; did not independently increase a mechanistic or therapeutic evidence grade.
[131] Wang et al., 2023	Review/contextual synthesis	DTP/stable resistance and adaptive-state context	Contextual evidence only	Defines DTP biology and distinguishes reversible tolerance from stable resistance.	Context only	Not applicable for grading in this review	Not independently appraised for grade	Not independently graded	Used for conceptual context; did not independently increase a mechanistic or therapeutic evidence grade.
[132] Rehman et al., 2021	Patient-derived CRC models with	Chemotherapy-induced DTP state	Graded mechanistic/therapeutic	CRC cells enter a reversible diapause-like DTP	Yes	Longitudinal chemotherapy, withdrawa	Patient-derived in vivo CRC models.	Moderate	Direct DTP evidence with functiona

	lineage/barcoding and in vivo treatment		evidence	state that survives chemotherapy and can regrow after withdrawal.		l and regrowth analysis.			l longitudinal validation in advanced patient-derived models; no prospective clinical validation.
[133] He et al., 2024	Review	DTP/plasticity and therapeutic vulnerabilities	Contextual evidence only	Context for drug-tolerant states and strategies targeting them.	Context only	Not applicable for grading in this review	Not independently appraised for grade	Not independently graded	Used for contextual synthesis and did not independently increase an evidence grade.
[134] Álvarez-Varela et al., 2022	Patient-derived CRC organoids and mouse models	Chemotherapy-induced DTP/relapse	Graded mechanistic/therapeutic evidence	MEX3A-positive persister cells survive chemotherapy and regenerate tumours after treatment.	Yes	Lineage tracing, chemotherapy exposure and regeneration/relapse assays.	Patient-derived organoids and in vivo models.	Moderate	Direct DTP evidence across complementary advanced models.
[135] Zhang et al., 2026	CRC experimental study	KRAS/EGFR-targeted resistance	Graded supporting evidence	Paneth-like lineage transition drives resistance to dual KRAS/EGFR targeting.	Partial/indirect	Functional state/pathway manipulation.	Advanced CRC models.	Indirect	Strong plasticity/resistance evidence in a targeted-therapy context.
[136] Bauzone et al., 2021	5-FU-sensitive and -resistant CRC cell models	5-FU/YAP-associated stemness	Graded mechanistic/therapeutic evidence	YAP-RAR/RXR crosstalk promotes stemness, self-renewal and 5-FU resistance.	Yes	YAP/RAR-RXR perturbation with stemness and treatment-response assays.	Cell models only.	Limited	Direct drug-specific functional evidence, but no complementary organoid/

Supplementary Table S1 and Supplementary Table S2

									in vivo validation.
[137] Jog et al., 2025	CRC DTPs, patient-derived organoids and in vivo models	DTP metabolic resistance	Graded mechanistic/therapeutic evidence	LPIN1-dependent lipid storage is a vulnerability of therapy-resistant/DTP CRC cells.	Yes	LPIN1 inhibition/statins with ferroptosis and treatment-response assays.	DTPs, PDOs and in vivo models.	Moderate	Direct DTP mechanism and therapeutic vulnerability validated across complementary models.
[138] Mzoughi et al., 2025	WNT-dependent CRC models including patient-derived tumoroids and mouse models	FOLFIRI-associated oncofetal plasticity	Graded mechanistic/therapeutic evidence	YAP/AP-1-driven oncofetal reprogramming creates a drug-tolerant state associated with FOLFIRI resistance.	Yes	State/pathway manipulation with chemotherapy-response testing.	Patient-derived tumoroids and mouse models.	Moderate	Direct chemotherapy-plasticity evidence across complementary advanced models.
[139] Alonso et al., 2026	Patient-matched/experimental CRC study	KRAS-inhibitor resistance	Graded supporting evidence	Concurrent genetic and non-genetic resistance mechanisms include adaptive state changes.	Partial/indirect	Longitudinal molecular/functional analysis.	Human matched biopsies/experimental models.	Indirect	Resistance evidence is direct for KRAS inhibition, not conventional cytotoxic chemotherapy.
[140] Ren et al., 2018	CAF-CRC models, human CRC specimens and in vivo models	TME/exosomal chemoresistance	Graded mechanistic/therapeutic evidence	CAF-derived exosomal H19 promotes stemness and chemoresistance through β-catenin signalling.	Yes	Exosome/H19/miR-141 manipulation with chemosensitivity and tumour-initiation assays.	Human CRC specimens plus in vivo validation; human samples support pathway/stromal expression but are not	Moderate	Direct functional non-cell-autonomous mechanism reproduced in vivo; human tissue is supportive but does not directly

Supplementary Table S1 and Supplementary Table S2

							treatment-response cohorts.		validate treatment resistance.
[141] Gonçalves-Ribeiro et al., 2016	CRC cells exposed to carcinoma-associated fibroblasts	5-FU/oxaliplatin TME resistance	Graded mechanistic/therapeutic evidence	CAFs alter CRC sensitivity to 5-FU and oxaliplatin.	Yes	CAF-conditioned/co-culture treatment-response testing.	Predominantly preclinical cellular evidence.	Limited	Direct TME-mediated chemotherapy evidence, but complementary mechanistic/in vivo validation is limited.
[142] Linares et al., 2023	Human residual CRC tissue plus CAF experimental models	Platinum-treated TME resistance	Graded mechanistic/therapeutic evidence	Long-term platinum accumulation in CAFs activates pro-tumour stromal programmes and supports treatment resistance.	Yes	Elemental imaging and CAF functional/mechanistic studies.	Residual human CRC after platinum therapy with complementary experimental models.	Strong	Treatment-exposed human tissue directly supports the stromal phenomenon alongside mechanistic experimental evidence.
[143] Wang et al., 2016	CRC experimental models	IL-6/STAT3 platinum response	Graded mechanistic/therapeutic evidence	IL-6/STAT3 signalling lowers sensitivity to platinum-containing therapy.	Partial	IL-6 inhibition with antitumour/treatment-response testing.	Preclinical evidence; carboplatin context is outside the principal CRC drugs reviewed.	Indirect	Supports cytokine-mediated platinum resistance conceptually, but the treatment context is not directly oxaliplatin/5-FU/irinotecan acquired resistance.
[144] Agorku et al	CRC CAF/immune	TME immune	Graded supporting	CAF NECTIN2 signalling	Partial/in	Functional immune-stromal	CRC TME models.	Indirect	Supports immunosuppressiv

Supplementary Table S1 and Supplementary Table S2

al., 2024	experimental study	suppression	evidence	suppresses effector T cells.	direct	experiments.			e TME biology, but not direct chemotherapy resistance.
[145] Tang et al., 2018	CRC cells, CAF/hypoxia models, PDXs and patient outcome data	Hypoxic TME chemoresistance	Graded mechanistic/therapeutic evidence	HIF-1α/TGF-β2-GLI2 signalling drives stemness and chemotherapy resistance in hypoxic CRC.	Yes	Genetic/pharmacological pathway inhibition reverses TME-mediated chemoresistance.	Patient relapse association and chemotherapy-responsive/resistant PDX models.	Strong	Concordant functional mechanism, patient outcome association and patient-derived in vivo validation.
[146] Xu et al., 2019	CRC cells, human CRC specimens and xenografts	Hypoxia-induced drug resistance	Graded mechanistic/therapeutic evidence	HIF-1α/miR-338-5p/IL-6 feedback drives hypoxia-mediated resistance.	Yes	miRNA/pathway perturbation and chemosensitivity assays.	Human CRC specimens show pathway correlations; xenografts provide direct oxaliplatin-sensitivity validation.	Moderate	Direct functional mechanism with complementary in vivo validation; human tissue supports pathway relevance but does not directly validate treatment resistance.
[147] Lotti et al., 2013	Matched human CRC specimens, CAF/CIC models and in vivo experiments	Chemotherapy-induced TME remodelling	Graded mechanistic/therapeutic evidence	Chemotherapy activates CAFs to maintain CRC-initiating cells via IL-17A.	Yes	CAF/IL-17A manipulation with self-renewal and tumour-growth assays.	Matched patient samples before/after cytotoxic treatment plus in vivo models.	Strong	Direct treatment-induced stromal mechanism with human treatment-exposed and in vivo validation.

Supplementary Table S1 and Supplementary Table S2

[148] Yin et al., 2025	CAF-CRC models, neoadjuvant-treated patient CAFs and in vivo models	Chemotherapy-induced CAF/DKK1 resistance	Graded mechanistic/therapeutic evidence	Chemotherapy induces CAF-derived DKK1, promoting immunosuppression and chemoresistance; DKK1 neutralisation improves 5-FU response.	Yes	p53/MEK-ERK/DKK1 perturbation and DKK1 neutralisation with chemotherapy.	Neoadjuvant-treated patient CAFs plus in vivo validation.	Moderate	Direct functional mechanism with treatment-exposed human stromal support and in vivo validation; the DKK1-neutralising therapeutic strategy remains preclinical.
[149] Calon et al., 2012	CRC stromal experimental/translational study	TGF-β stromal programme	Graded supporting evidence	TGF-β-driven stromal programmes support metastatic fitness.	Partial/indirect	Stromal pathway manipulation.	Human/preclinical CRC models.	Indirect	Relevant non-cell-autonomous biology, but not a direct chemotherapy-resistance mechanism.
[150] Hu et al., 2015	CRC cell/CSC models, patient-derived xenograft material and in vivo models	Fibroblast-exosome-mediated chemoresistance	Graded mechanistic/therapeutic evidence	Fibroblast-derived exosomes prime CRC stem-like cells and increase 5-FU/oxaliplatin resistance.	Yes	Exosome-transfer/depletion experiments with chemotherapy-response and tumour-growth assays.	Patient-derived xenograft CSCs and in vivo chemotherapy models.	Moderate	Direct functional non-cell-autonomous mechanism validated across complementary patient-derived and in vivo models; no direct clinical validation.

Supplementary Table S1 and Supplementary Table S2

[151] Deng et al., 2020	CAF-exosome/CRC cell and in vivo models with human tumour-stroma analysis	Oxaliplatin resistance	Graded mechanistic/therapeutic evidence	CAF-derived exosomal CCAL activates β-catenin and promotes oxaliplatin resistance.	Yes	CCAL/exosome perturbation with apoptosis, β-catenin and chemosensitivity assays.	Human CRC stromal expression plus in vivo validation; human tissue is not a treatment-response cohort.	Moderate	Direct functional mechanism reproduced in vivo; human tumour-stroma expression supports relevance but does not directly validate oxaliplatin resistance.
[152] Ning et al., 2021	CRC patient serum, resistant cells and mouse xenografts	Oxaliplatin/FOLFOX resistance and immune suppression	Graded mechanistic/therapeutic evidence	Tumour exosomal miR-208b promotes Treg expansion and oxaliplatin resistance.	Yes	Exosome/miR-208b-PDCD4 manipulation and Treg/treatment-response assays.	FOLFOX-treated patient serum plus in vivo xenograft validation.	Strong	Direct functional mechanism with human treatment-response biomarker and in vivo validation.
[153] Lee et al., 2020	Human translational study	CRC immune landscape	Contextual evidence only	Lineage-dependent immune organisation	Context only	Not applicable for grading in this review	Not independently appraised for grade	Not independently graded	Used for clinical, biological, methodological or background context; did not independently increase a mechanistic or therapeutic evidence grade.

Supplementary Table S1 and Supplementary Table S2

[154] Pelka et al., 2021	Human spatial study	CRC TME	Contextual evidence only	Spatial immune organisation	Context only	Not applicable for grading in this review	Not independently appraised for grade	Not independently graded	Used for clinical, biological, methodological or background context; did not independently increase a mechanistic or therapeutic evidence grade.
[155] Wu et al., 2022	Human CRC liver-metastasis single-cell and spatial transcriptomic study	Neoadjuvant chemotherapy-associated TME remodelling	Graded mechanistic/therapeutic evidence	Neoadjuvant chemotherapy is associated with substantial remodelling of the immune microenvironment in colorectal liver metastases, including macrophage-state changes and restoration of antitumour immune balance in responsive patients.	Yes	Single-cell RNA-seq and spatial transcriptomic profiling; no perturbational validation of the inferred mechanism.	Matched human colorectal liver-metastasis samples, including chemotherapy-treated responsive and non-responsive patients.	Limited	Direct human treatment-associated evidence, but the study is observational and does not establish causal functional resistance mechanisms.
[156] Qin et al., 2023	Human rectal cancer single-cell/spatial study	Neoadjuvant chemotherapy and CAF remodelling	Graded mechanistic/therapeutic evidence	Neoadjuvant chemotherapy reshapes CAF states and	Yes	Single-cell/spatial observational analysis.	Human paired/treatment-exposed tissue.	Limited	Direct human treatment-associated evidence,

Supplementary Table S1 and Supplementary Table S2

				tumour-microenvironment interactions.					but mechanistic causality is not established.
[157] Tie et al., 2016	Prospective/longitudinal clinical colon cancer cohort	Minimal residual disease biomarker	Graded mechanistic/therapeutic evidence	Post-treatment ctDNA detects residual disease and predicts recurrence.	Yes	Longitudinal clinical biomarker assessment.	Human clinical cohort.	Limited	Strong clinical association for residual disease, but this is a biomarker rather than a functional resistance-mechanism study.
[158] Schøler et al., 2017	Longitudinal clinical CRC cohort	ctDNA monitoring	Graded mechanistic/therapeutic evidence	Serial ctDNA can detect recurrent/evolving CRC after treatment.	Yes	Longitudinal clinical monitoring.	Human clinical cohort.	Limited	Clinically relevant longitudinal evidence without functional validation of a resistance mechanism.
[159] Avolio et al., 2026	Metastatic CRC patient-derived xenografts and tumoroids	FOLFIRI resistance	Graded mechanistic/therapeutic evidence	FOLFIRI-resistant mCRC models show distinct molecular/functional resistance states and targetable DNA-repair dependencies.	Yes	Functional resistance profiling and intervention in patient-derived models.	PDXs and patient-derived tumoroids; translationally human-derived but not prospective clinical validation.	Moderate	Direct FOLFIRI-resistance evidence across complementary patient-derived models.
[160] Guinney et	Molecular-classification study	CMS	Contextual evidence only	Consensus molecular subtypes	Context only	Not applicable for grading in	Not independently apprais	Not independently	Used for clinical, biological,

Supplementary Table S1 and Supplementary Table S2

al., 2015						this review	ed for grade	graded	methodological or background context; did not independently increase a mechanistic or therapeutic evidence grade.
[161] Ten Hoorn et al., 2022	Systematic review/meta-analysis	CMS	Contextual evidence only	Clinical value of CRC molecular subtypes	Context only	Not applicable for grading in this review	Not independently appraised for grade	Not independently graded	Used for clinical, biological, methodological or background context; did not independently increase a mechanistic or therapeutic evidence grade.
[162] Arqués et al., 2016	CRC targeted-therapy resistance study	PI3K/AKT inhibitor resistance	Graded supporting evidence	Tankyrase/Wnt inhibition reverses resistance to PI3K/AKT inhibitors.	Partial/indirect	Pathway inhibition and resistance assays.	Preclinical CRC models.	Indirect	Direct resistance biology in a targeted-therapy rather than cytotoxic-chemotherapy context.
[163] Song et al., 2018	Acquired 5-FU-resistant CRC cells, mouse models and chemotherapy-treated	5-FU/YAP resistance	Graded mechanistic/therapeutic evidence	YAP signalling is functionally required for acquired 5-FU resistance and	Yes	YAP knockdown with 5-FU sensitisation in vitro/in vivo.	Human chemotherapy-treated cohort plus mouse validation.	Strong	Direct acquired-resistance mechanism with concordant functional, in vivo and human

Supplementary Table S1 and Supplementary Table S2

	patient cohort			relates to relapse after 5-FU-based chemotherapy.					treatment-outcome evidence.
[164] Sakshaug et al., 2023	Systematic review	PDOs	Contextual evidence only	Predictive value of organoids	Context only	Not applicable for grading in this review	Not independently appraised for grade	Not independently graded	Used for clinical, biological, methodological or background context; did not independently increase a mechanistic or therapeutic evidence grade.
[165] Clara et al., 2020	Review	CSC pathways	Contextual evidence only	Clinical targeting of CSC-associated pathways	Context only	Not applicable for grading in this review	Not independently appraised for grade	Not independently graded	Used for clinical, biological, methodological or background context; did not independently increase a mechanistic or therapeutic evidence grade.
[166] Lu et al., 2024	CRC in vivo drug-delivery study	Notch-targeted strategy	Graded supporting evidence	Tumour-responsive Notch1 shRNA delivery suppresses CRC growth.	Partial/indirect	Functional Notch1 attenuation.	In vivo CRC models.	Indirect	Therapeutic proof-of-concept without direct acquired chemotherapy-resistance modelling.

Supplementary Table S1 and Supplementary Table S2

[167] Yap et al., 2025	Phase 1/2 solid-tumour trial	YAP/TEAD druggability	Graded supporting evidence	VT3989 demonstrates clinical YAP/TEAD pathway inhibition in solid tumours.	Partial/indirect	Clinical pharmacological intervention.	Human trial, not resistance-selected CRC.	Indirect	Clinical pathway-targeting evidence, but not CRC chemotherapy-resistance validation.
[168] Lombardo et al., 2011	CRC CSC and mouse study	Differentiation-based chemosensitisation	Graded supporting evidence	BMP4 differentiation increases CRC stem-cell response to chemotherapy.	Partial/indirect	BMP4 differentiation with chemotherapy-response testing.	In vivo mouse validation.	Indirect	Functional chemosensitisation evidence, but not an acquired-resistance model.
[169] Gao et al., 2025	Human NAC single-cell data, CRC cell models and animal models	Oxaliplatin/neoadjuvant chemoresistance	Graded mechanistic/therapeutic evidence	KLF5-PI3K/AKT signalling drives neoadjuvant oxaliplatin resistance and is targetable with PI3K inhibition.	Yes	Mechanistic KLF5/PI3K-AKT perturbation, drug screening and oxaliplatin combination testing.	Treatment-exposed human scRNA-seq plus animal validation.	Strong	Direct human treatment-state evidence with concordant mechanistic and in vivo validation.
[170] Herpers et al., 2022	Patient-derived organoid and in vivo study	EGFR×LGR5 targeted strategy	Graded supporting evidence	PDO screening identified petosemtamab/MCLA-158 as a tumour-selective therapeutic strategy.	Partial/indirect	Functional PDO screening and in vivo testing.	PDO and in vivo models.	Indirect	Strong therapeutic proof-of-concept, but not acquired cytotoxic chemoresistance.
[171] Baraibar et al., 2021	Review	MSS CRC immunotherapy	Contextual evidence only	Combination immunotherapy strategies	Context only	Not applicable for grading in this review	Not independently appraised for grade	Not independently graded	Used for clinical, biological, methodological or background context; did not independently

Supplementary Table S1 and Supplementary Table S2

									increase a mechanistic or therapeutic evidence grade.
[172] Hamid et al., 2024	Review	MSS metastatic CRC	Contextual evidence only	Immunotherapy context	Context only	Not applicable for grading in this review	Not independently appraised for grade	Not independently graded	Used for clinical, biological, methodological or background context; did not independently increase a mechanistic or therapeutic evidence grade.
[173] Shah et al., 2023	Randomised clinical trial	Mechanism-guided combination therapy	Contextual evidence only	Clinical outcome of napabucasin plus FOLFIRI-based treatment.	Context only	Randomised clinical treatment comparison	Human phase III trial	Not independently graded	Used to demonstrate that adding a putative resistance-targeting agent does not necessarily improve clinical outcome; not used to grade a specific acquired-resistance mechanism.
[174] Antoniotti et al., 2024	Randomised phase II clinical trial, four-year analysis	FOLFOXIRI/bevacizumab ± atezolizumab	Contextual evidence only	Updated overall-survival and exploratory biomarker outcomes from the	Context only	Randomised clinical treatment comparison	Human phase II trial; four-year follow-up	Not independently graded	Used for clinical treatment-selection context rather than to grade a specific acquired-

Supplementary Table S1 and Supplementary Table S2

				AtezoTRIBE trial.					resistance mechanism; overall survival was not the primary endpoint.
[175] Wen et al., 2023	Review	Single-cell sequencing	Contextual evidence only	Single-cell profiling in CRC	Context only	Not applicable for grading in this review	Not independently appraised for grade	Not independently graded	Used for methodological context.
[176] Alzamami, 2023	Review	Single-cell TME	Contextual evidence only	Immune-landscape implications	Context only	Not applicable for grading in this review	Not independently appraised for grade	Not independently graded	Used for methodological/contextual synthesis.
[177] Joanito et al., 2022	Human CRC single-cell and bulk transcriptomic study	CRC epithelial heterogeneity and molecular classification	Contextual evidence only	Single-cell profiling identifies intrinsic epithelial tumour states and refines bulk consensus molecular classification in CRC.	Context only	Not applicable for grading in this review	Large multi-cohort human CRC single-cell dataset with validation in bulk transcriptomic cohorts.	Not independently graded	Used to support molecular-classification and tumour-heterogeneity context rather than a specific acquired chemotherapy-resistance mechanism.
[178] Chen et al., 2026	Single-cell bioinformatic CRC resistance study with external datasets	Oxaliplatin-resistant subpopulations	Graded mechanistic/therapeutic evidence	An EpC2 epithelial state is associated with oxaliplatin resistance and suggests candidate therapies.	Yes	Computational identification and external dataset validation; no direct drug perturbation.	Single-cell and resistant-cell-line datasets.	Limited	Direct resistance profiling, but functional causal validation of the proposed state/therapy is absent.
[179] Nojima et al., 2025	Patient-derived colorectal organoids with scRNA-	DTP identification/targeting	Graded mechanistic/therapeutic evidence	Machine-learning-defined DTP populations can be	Yes	Organoid viability testing of predicted candidate drugs.	Patient-derived organoid model; no in	Limited	Functional patient-derived evidence, but validatio

Supplementary Table S1 and Supplementary Table S2

	seq, machine learning and drug testing			targeted by candidate drug combinations in malignant organoids.			vivo/clinical validation.		n remains within a single model class.
[180] Carranza et al., 2024	Review	Spatial biology	Contextual evidence only	Spatial profiling in CRC	Context only	Not applicable for grading in this review	Not independently appraised for grade	Not independently graded	Methodological/contextual source only.
[181] Guo et al., 2024	Review	Spatial transcriptomics	Contextual evidence only	Spatiotemporal profiling context	Context only	Not applicable for grading in this review	Not independently appraised for grade	Not independently graded	Methodological/contextual source only.
[182] Xiao et al., 2024	Human CRC single-cell and spatial transcriptomic study	Spatial tumour heterogeneity and tumour–microenvironment interactions	Contextual evidence only	Integrated single-cell and spatial transcriptomics maps malignant-cell heterogeneity and spatial tumour–stromal/immune interactions in CRC.	Context only	Not applicable for grading in this review	Human CRC tumour-normal-blood samples profiled by scRNA-seq with spatial transcriptomic integration.	Not independently graded	Directly demonstrates integrated spatial and single-cell profiling in CRC, but does not test a specific acquired chemotherapy-resistance mechanism.
[183] Gupta et al., 2019	Review	Colon CSCs	Contextual evidence only	CSC therapeutic targeting	Context only	Not applicable for grading in this review	Not independently appraised for grade	Not independently graded	Secondary contextual source only.
